# Association between the potential distribution of *Lutzomyia longipalpis* and *Nyssomyia whitmani* and leishmaniasis incidence in Piauí State, Brazil

**DOI:** 10.1371/journal.pntd.0011388

**Published:** 2023-06-05

**Authors:** Raimundo Leoberto Torres de Sousa, Thais de Araujo-Pereira, Anangela Ravena da Silva Leal, Simone Mousinho Freire, Cleanto Luiz Maia Silva, Jacenir Reis dos Santos Mallet, Mauricio Luiz Vilela, Silvia Alcântara Vasconcelos, Régis Gomes, Clarissa Teixeira, Constança Britto, Daniela de Pita Pereira, Bruno Moreira de Carvalho

**Affiliations:** 1 Laboratório de Biologia Molecular e Doenças Endêmicas, Instituto Oswaldo Cruz—Fiocruz, Rio de Janeiro, Brasil; 2 Laboratório de Zoologia e Biologia Parasitária, Universidade Estadual do Piauí, Teresina, Piauí, Brasil; 3 Laboratório Interdisciplinar de Vigilância Entomológica em Díptera e Hemíptera, Instituto Oswaldo Cruz—Fiocruz, Rio de Janeiro, Brasil; 4 Escritório Regional Fiocruz-Piauí, Teresina, Piauí, Brasil; 5 Laboratório de Vigilância e Biodiversidade em Saúde, Universidade Iguaçu, Nova Iguaçu, Rio de Janeiro, Brasil; 6 Departamento de Biotecnologia Fiocruz-Ceará, Eusébio, Ceará, Brasil; 7 Climate and Health Program, Barcelona Institute for Global Health, Barcelona, Spain; Instituto Oswaldo Cruz, BRAZIL

## Abstract

**Background:**

Leishmaniases are vector borne diseases caused by *Leishmania* spp. parasites transmitted by female sandflies (Diptera: Psychodidae) whose geographic distribution is influenced by environmental factors. Among the main tools for studying the distribution of vector species, modeling techniques are used to analyze the influence of climatic and environmental factors on the distribution of these insects and their association with human cases of the disease.

**Methodology/Principal findings:**

Here, we used a multiscale ecological niche modeling approach to assess the environmental suitability of sandfly vectors of the etiological agents of Visceral (VL) and American Cutaneous Leishmaniasis (ACL) in Piauí state, northeastern Brazil, and then evaluated their relationship with human disease incidence. For this, we obtained the geographic coordinates of the vector species *Lutzomyia longipalpis* and *Nyssomyia whitmani* through literature review, online databases and unpublished records. These data were used for the development of predictive models of the distribution of both sandflies species based on climatic and environmental variables. Finally, the environmental suitability for the presence of these vectors was compared with the incidence of both the diseases at the municipality level. The final models for each sandfly species showed good predictive powers with performance metric values of 0.889 for *Lu*. *longipalpis* and 0.776 for *Ny*. *whitmani*. The areas with greater environmental suitability for the presence of these species were concentrated in the central-north region of Piauí and coincide with the location of those municipalities presenting higher incidences of VL and ACL, situated in the central-north and extreme north of the state, respectively. The south and southeast regions of Piauí state have low incidence of these diseases and presented low environmental suitability for the presence of both vectors.

**Conclusions/Significance:**

We discuss how predictive modeling can guide entomological and epidemiological surveillances and recommend an increased supervision and control activities in Teresina (capital of the state of Piaui), Altos and Pedro II, in addition to other municipalities with similar social and environmental characteristics.

## Author summary

Sandflies are insects that actively participate in the transmission cycles of leishmaniasis and other diseases, and these invertebrate vectors are frequently found in areas with hot and humid climates. However, in addition to the climate, there are several factors that contribute to the occurrence of sandfly vectors, such as, the vegetation type, altitude, and land use and cover. Understanding which environmental variables are important for the presence of sandflies can help us to understand how these insects are distributed and overall, how this affects the transmission of the etiological agents of leishmaniasis. In this study, we performed a set of ecological models where environmental and climatic variables were combined in multiple scales and correlated with the presence of two sandflies species, *Lutzomyia longipalpis* and *Nyssomyia whitmani*, vectors of parasites causing visceral and cutaneous leishmaniasis, respectively. We then combined this information with human disease data to assess potential risk areas in the state of Piauí, northeast Brazil. The resulting maps showed that municipalities of the central-north region of the state have favorable environment for the occurrence of these vectors in addition to higher incidence of leishmaniasis in humans. We thus recommend that control activities should be focused on this region of the state.

## Introduction

Vector-borne diseases are widespread across the globe and affect millions of people living in endemic areas [1]. The increase in the number of cases is closely related to climate and environmental factors, since insect vectors are vulnerable to the climate and consequently to changes resulting from global warming [2].

Among the main vector-borne diseases, leishmaniasis is a neglected tropical disease that has higher incidence following months with high rainfall [3]. Caused by protozoa of the genus *Leishmania* Ross, 1903, the main disease clinical manifestations in the American continent can be classified as Visceral Leishmaniasis (VL), being *Leishmania (Leishmania) infantum chagasi* Nicolle, 1908 and Chagas, 1937 the solely etiological agent and American Cutaneous Leishmaniasis (ACL), caused mostly by *Leishmania (Viannia) braziliensis* Vianna, 1911, although many other species are involved in complex transmission cycles [4,5].

Sandflies are insects of the order Diptera, family Psychodidae and subfamily Phlebotominae responsible for the transmission of several pathogens [6], among them, the unicellular protozoan of the genus *Leishmania* [7]. Globally, the sandflies are divided into 1,047 taxa (species or subspecies) with 532 of them occurring only in the Americas [6]. In Brazil, several species of medical importance are found, such as *Lutzomyia longipalpis* (Lutz & Neiva, 1912); *Nyssomyia whitmani* (Antunes & Coutinho, 1939); *Ny*. *intermedia* (Lutz & Neiva, 1912); *Ny*. *umbratilis* (Ward & Fraiha, 1977); *Psychodopygus wellcomei* Fraiha, Shaw & Lainson, 1971; *Bichromomyia flaviscutellata* (Mangabeira, 1942) and *Migonemyia migonei* (França, 1920) [6].

To verify vector competence and to incriminate sandfly species as vectors of *Leishmania*, some conditions must be fulfilled as established by Killick-Kendrick (1990), as for instance the spatial association between potential vectors and human leishmaniasis cases [8]. Other additional and complementary elements are the studies of vector competence and experimental infection in laboratory conditions [9]. Moreover, there are several tools that can suggest associations between sandflies and *Leishmania spp*. of the same or different transmission cycles, being the spatial distribution maps of the species involved in the epidemiological triad (parasite/vector/host), the most used strategy [10,11].

In northeastern Brazil, an unusual close relationship between vector and parasite can be observed in areas of the immediate region of Teresina (capital of Piauí state), where it is observed high incidence of ACL [12] and low density of *Ny*. *whitmani* sandflies, the species considered the main vector of *L*. *braziliensis* in Brazil [13]. Despite the low density of *Ny*. *whitmani*, this region has high abundance of *Lu*. *longipalpis* which is the main vector of *L*. *infantum chagasi* (the etiological agent of VL), raising questions about the epidemiological importance of other vector species in a local transmission cycle of leishmaniasis [14].

The geographic distribution of both *L*. *braziliensis* and *L*. *infantum chagasi* is associated to climatic and environmental factors related to their evolutionary history and interaction with their vectors and hosts [15]. These elements are structured in space across multiple spatial scales, and affect each species involved in leishmaniasis transmission cycles. Climate shapes the distribution of each species at larger scales, and as it narrows down to regional levels, the land use and habitat features gain more importance while biotic interactions control the microscale distributions [16]. In this multiscale scenario, suitable abiotic and biotic conditions determine the range of each species, and the overlaps in space and time can strengthen the relationship between the etiological agent and other host and vector species.

According to previous studies, the DNA of *L*. *braziliensis* has been detected in several vector species of the *Nyssomyia* and *Migonemyia* genera, with emphasis on *Ny*. *whitmani* and *Migonemyia migonei* [17,18]. The latter, although implicated in the notification of ACL cases [19,20], has already been incriminated as a competent vector for the transmission of *L*. *infantum chagasi* in the municipality of Baturité, state of Ceará, northeastern Brazil [9]. Moreover, other associations between sandflies and *Leishmania* spp. from different transmission cycles can occur in distinct regions of northeast of Brazil, such as *Leishmania (Leishmania) amazonensis* and *Lutzomyia longipalpis* in the municipality of Caxias, Maranhão state [21], mainly due to the impact of climatic factors and environmental changes on the distribution of vectors [22].

By assessing overlaps in the potential distribution areas of vectors and human disease, it is possible to identify possible leishmaniasis foci. In the state of Piauí, where regional differences in vector abundance lead to questions about the relevance of different sandfly species in both, ACL and VL transmission cycles, this approach has never been applied. So, this study was carried out aiming to analyze the influence of climatic and environmental factors on the distribution of sandfly species and further association with the location of human cases. Modelling techniques were used to assess this association and to predict suitable areas for the occurrence of vectors. By overlaying multiple combinations of the predicted distributions data of vectors and human disease, we further explore the local relationships between *Ny*. *whitmani* and *Lu*. *longipalpis* with ACL and VL notifications in the state of Piauí, Brazil.

## Methods

### Study area

The state of Piauí is located in the Northeast of Brazil (7°25′12″ S, 42° 54′ 0″ W) and has a territorial area of 251,755 km^2^ and an estimated population of 3,289,290 inhabitants, with a population density of 12.40 individuals/km^2^ [23]. It borders the states of Tocantins, Maranhão, Bahia, Ceará and Pernambuco. With 224 municipalities, the state is organized into 19 immediate geographic regions, which in turn are grouped into 6 intermediate geographic regions [24] (S1–S4 Figs).

**Fig 1:**
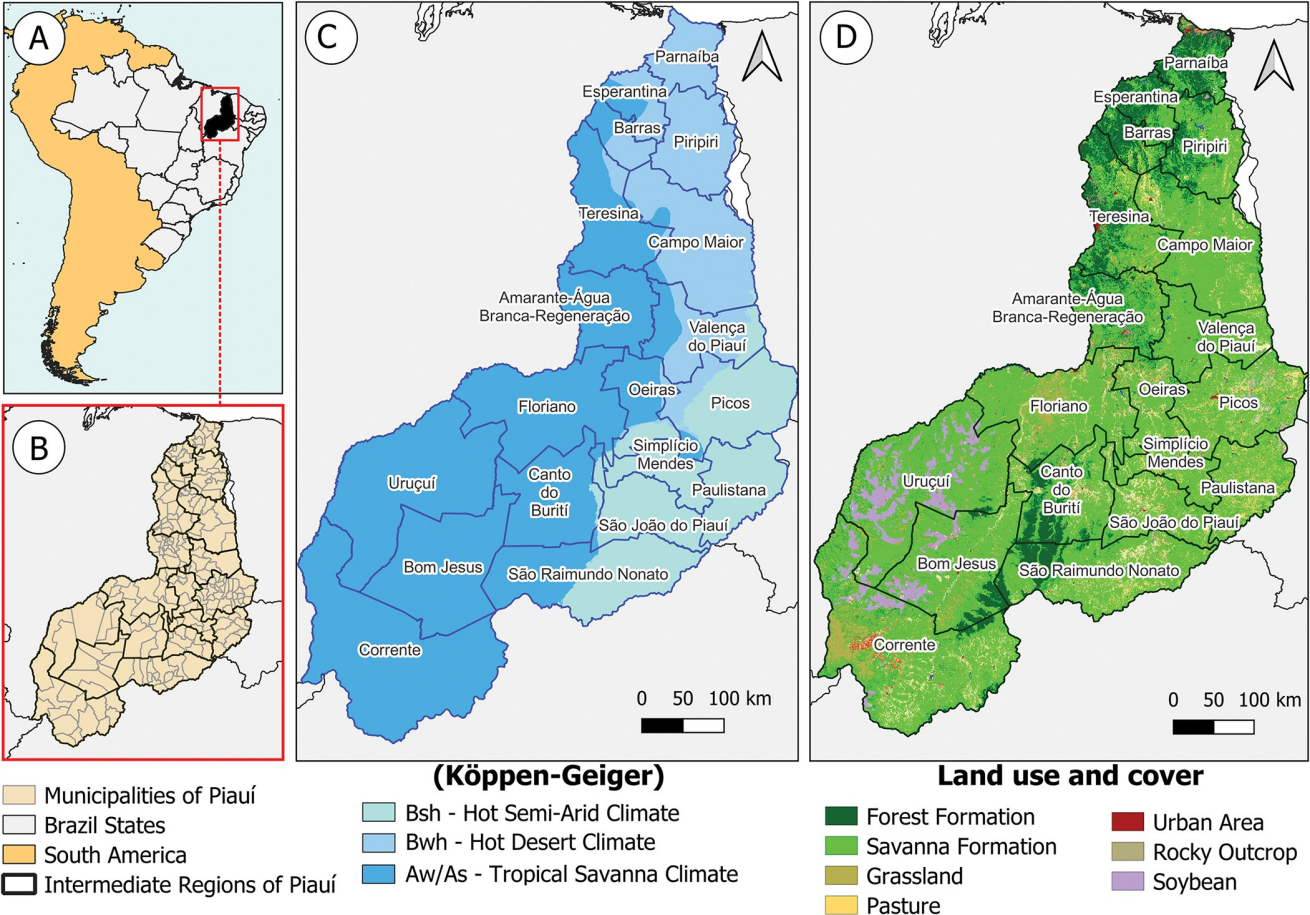
**Maps of Piauí representing: A.** South America with emphasis on the state of Piauí, Brazil. **B.** Municipalities and Intermediate geographic regions of Piauí. **C.** Climate classification map according to Köppen-Geiger showing the Tropical Savanna (Aw), hot semi-arid (Bsh) and hot desert (Bwh) climates. **D.** Land use / land cover and vegetation. Software used: QGIS; Source: MapBiomas. Available at: https://mapbiomas.org/colecoes-mapbiomas-1?cama_set_language=pt-BR and IBGE (Brazilian Institute of Geography and Statistics). Available at: https://www.ibge.gov.br/geociencias/informacoes-ambientais/climatologia/15817-clima.html?=&t=downloads.

Due to its territorial extension, Piauí presents regional climatic diversity. According to the Köppen-Geiger classification, most of the state territory has a tropical savanna climate (Aw/As) with temperature variation between 25 and 27°C and rainfall of 700 mm to 1.200 mm annually. Hot semi-arid climate (Bsh), is predominant only in the southeastern region of the state where the rainy season registers low rainfall of 600mm/year; the dry season extends up to 8 months where temperatures vary between 24 and 40°C. Hot desert climate (Bwh) is found in the northeast region with precipitation lower than 500 mm [25] (Fig 1).

Piauí has great vegetation diversity that varies according to its characteristics and physical aspects. The state covers a large transition area between the Caatinga biome—a kind of savanna, and the regional vegetation formed by the Cocais forest—a kind of palm forest. The vegetation is composed mostly of savanna formations and small forest areas, in addition to large areas of agriculture and livestock (Fig 1).

### Multiscale ecological niche modeling approach

**Fig 2:**
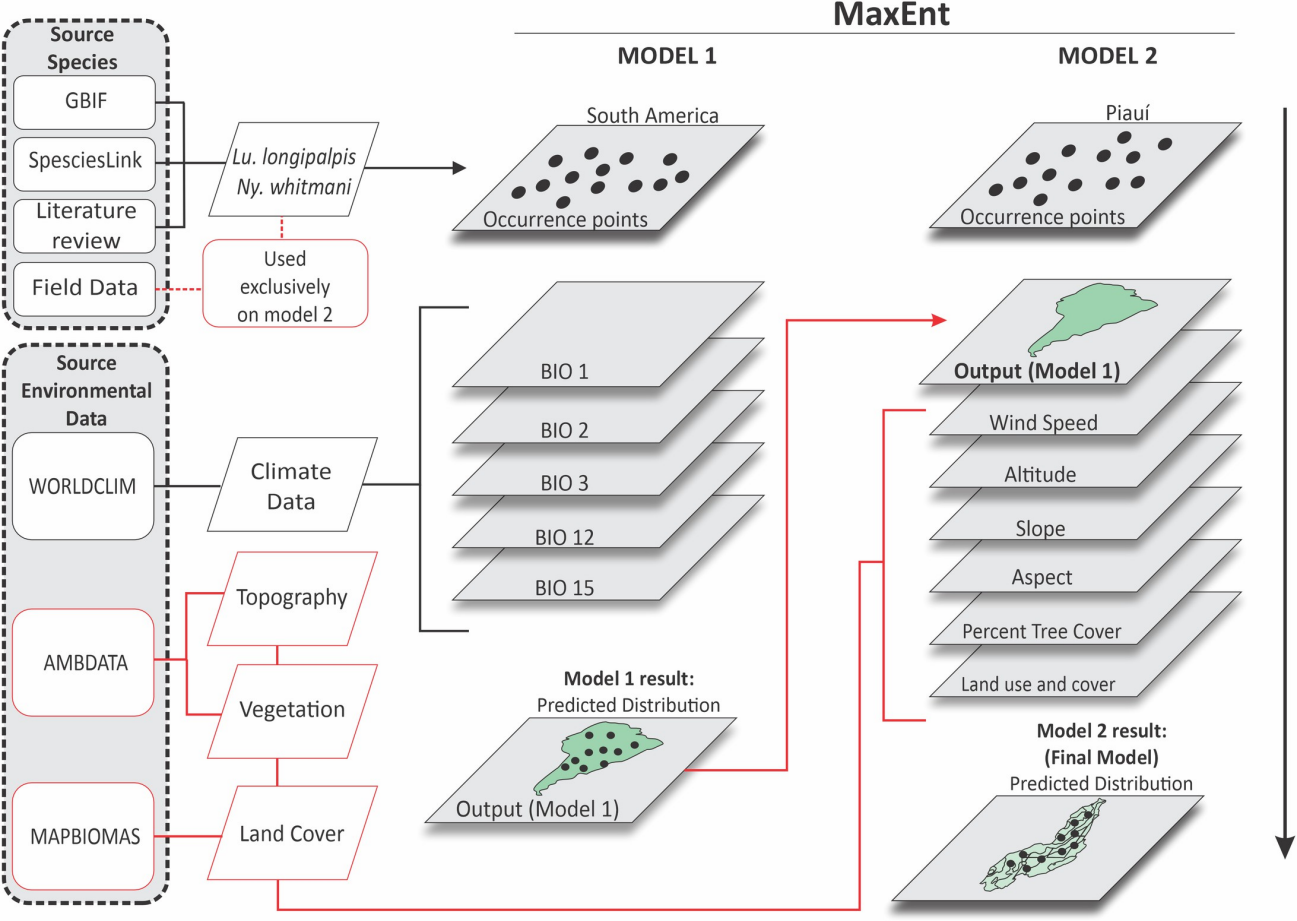
Scheme of the multiscale ecological niche modeling approach. GBIF: Global Biodiversity Information Facility; WORLDCLIM: World Climate; AMBDATA: environmental data; BIO: Bioclimatic; MaxEnt: Maximum Entropy.

To model the ecological niche and predict the spatial distribution of the vector species *Ny*. *whitmani* and *Lu*. *longipalpis*, climatic and environmental variables were analyzed according to their scale domain (resolution). A two-step multiscale approach was carried out (Fig 2).

The first model (model 1) used the geographic coordinates of the vector species across their full known ranges in South America and raster layers of climatic factors with a spatial resolution of 2.5 arc-minutes (approximately 5 km^2^). The climatic variables were used on this scale due to their relation with the distribution of species in large territorial extensions.

In the second stage, the result of model 1 was cropped to the territorial dimensions of Piauí and used as an input variable of model 2, with other non-climatic variables, such as topography and land use/cover, in higher resolution (approximately 1 km^2^). These variables are considered more determinant in limiting species distribution at the regional scale [16] (Fig 2).

The ecological niche model of the species was then overlaid with the incidence of VL and ACL in Piauí in order to analyze the relationship between the environmental suitability of the species and the diseases occurrences, and to identify possible risk locations.

### Vector species data

An integrative review was carried out using the PRISMA method (Preferred Reporting Items for Systematic Reviews and Meta-Analyses) [26]. The search terms were: “longipalpis”, “whitmani”, “fauna” and “Leishmaniasis” in addition to the use of the Boolean operator “OR”. Two databases were searched: The Scientific Electronic Library Online—SciELO and the National Library of Medicine—NIH-PubMed. For the selection of articles, the following inclusion criteria were used: language (English and Portuguese) and period of publication (between 1972 and 2020). For the exclusion criteria: duplicate articles and/or articles that do not specify the origin of specimens found at any departmental level (e.g., neighborhood, county, state, or country).

After searching and selecting articles, documents were analyzed for occurrence records of *Lu*. *longipalpis* and *Ny*. *whitmani*. Extracted data were organized in a spreadsheet with information on capture locations (country, state/province/department, district/municipality, and geographic coordinates). For articles that did not inform the exact latitude and longitude of the capture sites, the coordinates were obtained through Google Earth (https://earth.google.com/) using the center of the neighborhood, locality or city/municipality center. According to the methodology by Falcão de Oliveira et al, 2018 [27], after assigning latitude and longitude coordinates to each point of vector occurrence, these parameters were classified into three levels of spatial precision: High level, when the capture site coordinates were available in the original source; Medium level, when the coordinates of the locality or neighborhood were obtained from Google Earth; and Low level, when the coordinates of the municipality administrative center were obtained. Records with information only at the state level or higher (e.g. country, region, biome) were removed from the database.

To increase the number of records of selected vector species and to calibrate model 1, data from the SpeciesLink network [28] and Global Biodiversity Information Facility—GBIF [29] were obtained using the search terms: “*Nyssomyia whitmani”* and “*Lutzomyia longipalpis”*. The search was performed using two filters: country of collection (Brazil) and location (including coordinates). From the variables available in GBIF and SpeciesLink, the following were retained: species name, geographic coordinates, state and municipality of capture.

Additional unpublished vector species records from other studies of the group held in Piauí in the municipalities of Teresina, Altos, Pedro II and Oeiras (Raimundo Sousa, personal communication) were included in model 2, due to high spatial precision.

The species records database from the multiple sources contained 8,092 records of *Ny*. *whitmani* and 3,603 records of *Lu*. *longipalpis*. After taxonomic review (standardization of nomenclatures) and exclusion of duplicates, the final database used in the models had 297 records of *Ny*. *whitmani* and 218 records of *Lu*. *longipalpis*.

### Climatic and environmental data

For model 1, a set of five bioclimatic variables with low multicollinearity was selected from the original set constituted by 19 bioclimatic variables retrieved from WorldClim (https://www.worldclim.org/) (Table 1). Pixel values from the 19 variables were extracted using the Point Sampling Tool plugin of QGIS (https://qgis.org/en/site/), and analyzed using Spearman’s non-parametric correlation test to select only variables with a correlation lower than 0.7.

**Table 1:** Variables selected for modeling the ecological niche of *Lu*. *longipalpis* and *Ny*. *whitmani* vectors in the state of Piauí.

Category	Name	Description	SR Native	SR Standardized	Model	Source
Climate variables	BIO1	Annual Average Temperature	~ 5 Km^2^	Native resolution	Model 1 (South America)	WORLDCLIM
Climate variables	BIO2	Average Day Range average of the month (max temperature—min temperature)	~ 5 Km^2^	Native resolution	Model 1 (South America)	WORLDCLIM
Climate variables	BIO3	Isothermal (average daily variation / annual average temperature) (×100)	~ 5 Km^2^	Native resolution	Model 1 (South America)	WORLDCLIM
Climate variables	BIO12	Annual Precipitation	~ 5 Km^2^	Native resolution	Model 1 (South America)	WORLDCLIM
Climate variables	BIO15	Precipitation Seasonality (coefficient of variation)	~ 5 Km^2^	Native resolution	Model 1 (South America)	WORLDCLIM
Climate variables	Climatic suitability (Model 1 output)	Predicted climatic suitability from model 1	~ 5 Km^2^	~ 1 Km^2^	Model 2 (Piauí)	WORLDCLIM
Climate variables	Wind speed	Wind speed measured in (m/s-1)	~ 5 Km^2^	~ 1 Km^2^	Model 2 (Piauí)	WORLDCLIM
Topography	Altitude	Vertical distance between the top of an area in relation to the sea level	~ 900 m^2^	~ 1 Km^2^	Model 2 (Piauí)	AMBDATA
Topography	Slope	Slope of the land surface in relation to the horizontal line	~ 900 m^2^	~ 1 Km^2^	Model 2 (Piauí)	AMBDATA
Topography	Aspect	Direction of slope variation that makes up terrain surface exposure geometry in relief scheme representations	~ 900 m^2^	~ 1 Km^2^	Model 2 (Piauí)	AMBDATA
Vegetation	Percentage of tree cover	Forest cover indicator for an area	~ 1 Km^2^	~ 1 Km^2^	Model 2 (Piauí)	AMBDATA
Land cover	Land use and cover	Land cover ranging from natural vegetation to changes directed to agriculture and urban structures	~ 30 m^2^	~ 1 Km^2^	Model 2 (Piauí)	MAPBIOMAS

SR: spatial resolution

For model 2, six environmental variables were used in addition to the result of model 1: wind speed (m/s-1), altitude, slope, aspect, percentage of tree cover, land use and cover (Table 1). Wind speed and altitude were also downloaded from the WorldClim database. The percentage of tree cover, slope and aspect were obtained from AMBDATA (http://www.dpi.inpe.br/Ambdata/index.php). Land use and cover data were obtained through MAPBIOMAS (https://mapbiomas.org), a yearly database of land use and cover categories based on Landsat mosaics, from which the data of 2020 were downloaded.

Model 1 (climatic suitability) was run in the native spatial resolution of the bioclimatic variables (2.5 arc-min). For model 2 (environmental suitability), as each variable presented different native spatial resolution (between ~30 m^2^ to ~5 km^2^), QGIS was used to standardize pixels, resampling all rasters to ~1 km^2^, including the climatic suitability (Table 1).

### Model parameters

The Maxent method is based on the maximum entropy approach for making species distributions. From a set of environmental grids and georeferenced occurrence coordinates, the generated model is performed by the MaxEnt software version 3.4.4 and expresses a probability distribution where each grid cell has a predicted suitability of conditions for the analyzed species [30,31]. MaxEnt predicts the environmental suitability of each pixel of the study area for the occurrence of the vector species, based on a set of locations where the species occur and a background sample of the environmental variables [32,33]. We used 10,000 background points and a convergence limit of 0.00001 with 500 iterations. Ten replicates of models for each species were run and the resulting mean of individual outputs was used.

The models generated by MaxEnt were evaluated by the AUC (Area Under the ROC Curve). This metric ranges from 0 to 1. When the result is less than or equal to 0.5, the model has low predictive power. Results between 0.5 and 1 indicate sufficient predictive power model. Thus, to consider the results, the following criteria were adopted: models with AUC values below 0.6 were considered of low predictive power; models with AUC between 0.7 and 0.8 were considered to have good predictive power; models with AUC between 0.8 and 0.9 were considered very good predictive power; and models with AUC values between 0.9 and 1.0 were considered excellent. To keep only the best performing models, we discarded all model replicates with AUC values below 0.8.

To assess individual variable contributions to the final models, the *jackknife* test present in the MaxEnt software was also ran. This test excludes one variable at a time, keeping the others during model processing.

### Environmental suitability maps

The models generated by MaxEnt were imported into the QGIS software to build maps of climate (model 1) and environmental (model 2) suitability; these were converted from ASCll to raster format using the WGS-84 Reference System. To make environmental suitability comparable with human disease data, model outputs were aggregated at the municipal level. For each analyzed municipality of Piauí, the mean of all pixel values within its borders was calculated. From this mean environmental suitability value, the municipalities were classified, for each vector species, as: unsuitable (below 0.1), medium suitability (0.1 to 0.4), high suitability (0.5 to 0.75), and excellent suitability (above 0.75).

### VL and ACL human cases in Piauí

Data on leishmaniasis human cases in Piauí were obtained from the Information Technology Department of the Brazilian Unified Health System (DATASUS) through SINAN [12]. The search included the municipalities of Piauí, tabulated one at a time, prioritizing those with the highest register of new simultaneous cases of VL and ACL and the period referring to confirmed cases between 2009 and 2020. Confirmed cases were compiled according to the number of affected municipalities and grouped into four study periods: 2009 to 2011, 2012 to 2014, 2015 to 2017, and from 2018 to 2020.

The population of each municipality in Piauí was obtained through data from the Brazilian Institute of Geography and Statistics–IBGE (https://www.ibge.gov.br/cidades-e-estados/pi.html) to calculate the incidence of the diseases. The generated maps of ACL and VL incidence were designed using QGIS version 3.10.14, applying the Jenks classification to determine the incidence rates. The incidence of diseases in the municipalities was calculated for each study period by the following equations:

$$IM\;=\;\frac{mACL}{mp}\times 100,000\;IM\;=\;\frac{mVL}{mp}\times 100,000$$

Where:

IM = average incidence

*mACL* = average number of ACL cases in the affected municipalities

*mVL* = average number of VL cases in the affected municipalities

*mp* = estimated population

### Association of potential vector distribution and the incidence of VL and ACL in the state of Piauí

To test the correlation between suitability for the vectors and incidence of diseases, a Spearman correlation test was performed. For the association it was necessary to normalize these two variables on the same scale. As the suitability interval presents results between 0 and 1, the incidence of VL and ACL was standardized for this same interval according to the equations:

$$Ip=\frac{iVL-min(iVL)}{\max\left(iVL\right)-min(iVL)}\;Ip=\frac{iACL-min(iACL)}{\max\left(iACL\right)-min(iACL)}$$

Where:

*Ip =* standardized incidence

*iVL =* mean VL incidence

*iACL =* mean ACL incidence

*min =* minimum value of average incidence

*max =* maximum value of average incidence

## Results

### Ecological niche models of vector species

According to bibliographic review and the search at GBIF and other sources, between 1972 and 2020, there were reports of sandflies in 23 municipalities in Piauí. The results of model 1, for South America, showed AUC values for *Lu*. *longipalpis* and *Ny*. *whitmani* equal to 0.917 and 0.844 respectively, suggesting that the generated models had very good predictive power. The areas with the greatest climatic suitability for *Lu*. *longipalpi*s were concentrated in the northeast region of Brazil and those for *Ny*. *whitmani* were located in the southeastern region of the country (S5 Fig).

**Fig 3:**
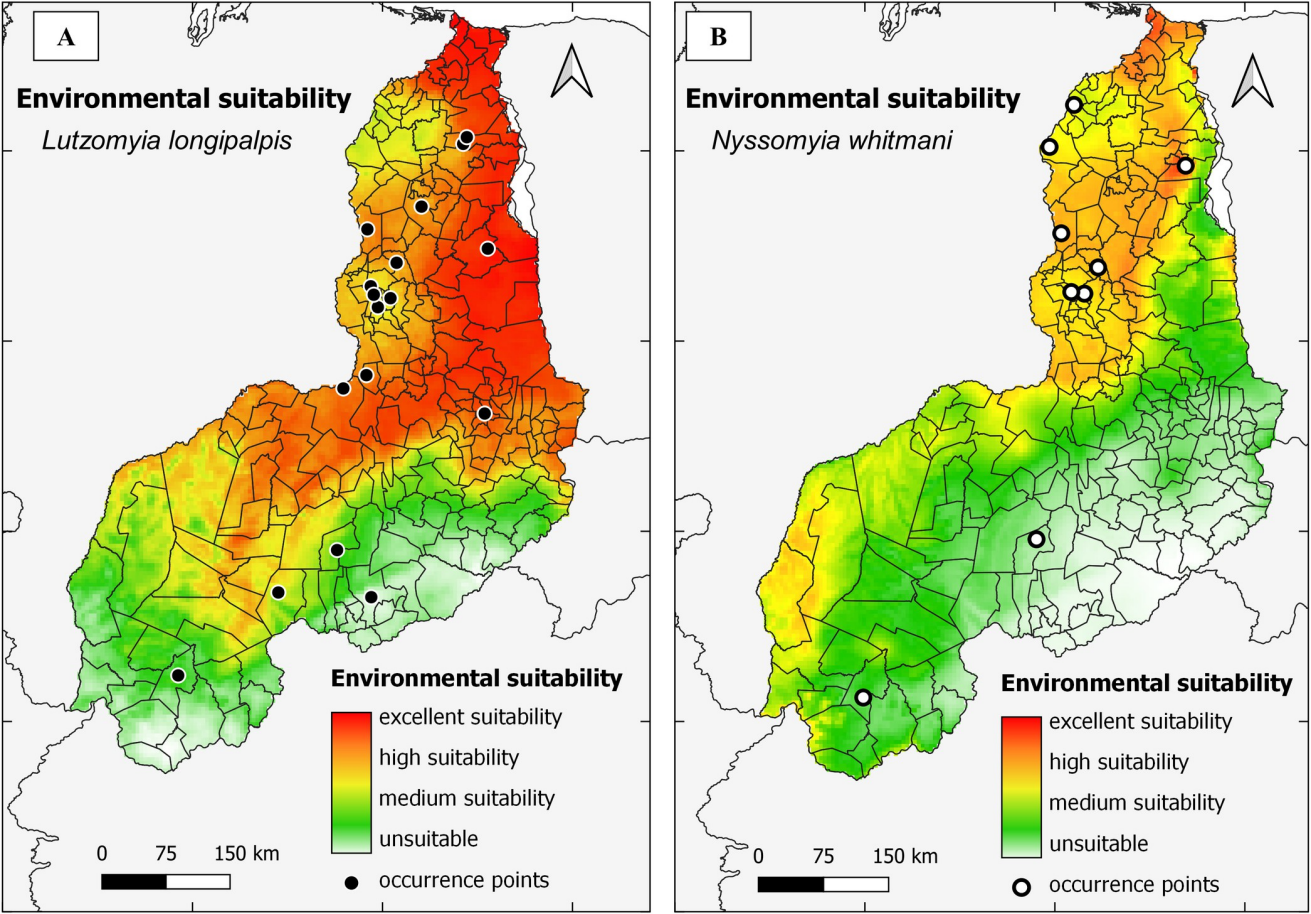
Environmental suitability of sandfly vectors of the causative agents of Visceral Leishmaniasis and American Cutaneous Leishmaniasis in the state of Piauí predicted by model 2. A. Environmental suitability for *Lu*. *longipalpis*. B. Environmental suitability for *Ny*. *whitmani*.

After running the final model (model 2), the AUC value for *Lu*. *longipalpis* was 0.889 (very good predictive power), whereas the AUC value for *Ny*. *whitmani* was 0.776 (good predictive power). The areas with the greatest environmental suitability for these species were concentrated in the north region of Piauí, being *Ny*. *whitmani* more directed to the extreme north (Fig 3).

Of the surveyed occurrence points, 70.59% presented low spatial precision for the occurrence of *Lu*. *longipalpis* and 11.76% had high precision. As for the occurrence of *Ny*. *whitmani*, 77.78% of the points were obtained with low spatial precision and 22.22% were registered with high precision (S6 Fig).

According to the predictive modeling (Fig 3), the intermediate regions of Teresina and Parnaíba (S1 Fig) presented excellent environmental suitability for the presence of *Lu*. *longipalpis* in all municipalities of both regions (65 in the intermediate region of Teresina and 30 municipalities in Parnaíba). For *Ny*. *whitmani*, it was observed high suitability in 51 municipalities (78.46%) of the intermediate region of Teresina; in the region of Parnaíba, 22 municipalities (73.33%) satisfied the environmental status as high and excellent for this species (Figs 3, S1, and S2 and S1 Table).

With the exception of the immediate region of Floriano (S1 Fig), which obtained high suitability for *Ny*. *whitmani* and high/excellent for *Lu*. *longipalpis* (Fig 3 and S1 Table), all other areas showed medium suitability for both species, with the presence of unsuitable areas for both of them in the municipalities composing the intermediate region of São Raimundo Nonato (Figs S1 and 3 and S1 Table).

Of all 224 municipalities in Piauí, 177 (79.02%) showed high and excellent suitability for the occurrence of *Lu*. *longipalpis* while 78 municipalities (34.82%) revealed high and excellent suitability for the presence of *Ny*. *whitmani*. In 64 (28.57%) municipalities it was observed high or excellent suitability for *Lu*. *longipalpis* and *Ny*. *whitmani* simultaneously. Regarding only the municipalities of Piauí displaying excellent environmental suitability for the presence of each sandfly species, it was observed 29 out of 224 (12.95%) for *Lu*. *longipalpis* and only 7 (3.13%) for *Ny*. *whitmani* (S1 Table).

Land use and cover together with climatic suitability were the variables that most contributed to the predicted environmental suitability of both species, *Lu*. *longipalpis* (61.4% and 31.5%, respectively) and *Ny*. *whitmani* (74.7% and 20.2%, respectively) (Table 2).

**Table 2:** Percent contribution of each variable on the environmental suitability. Influence of seven environmental variables on the models of *Lutzomyia longipalpis* and *Nyssomyia whitmani*, in the state of Piauí—Brazil.

Environmental Variables	*Lu*. *longipalpis* (%)	*Ny*. *whitmani* (%)
Altitude	1.7	1.3
Slope	0.7	2.3
Aspect	0.4	0.3
Climatic Suitability (model 1 output)	31.5	20.2
MODIS—Percent Tree Cover	1.3	1.0
Land use and cover	61.4	74.7
Wind Speed	3.0	0.2

MODIS, MODerate-resolution Imaging Spectroradiometer

**Fig 4:**
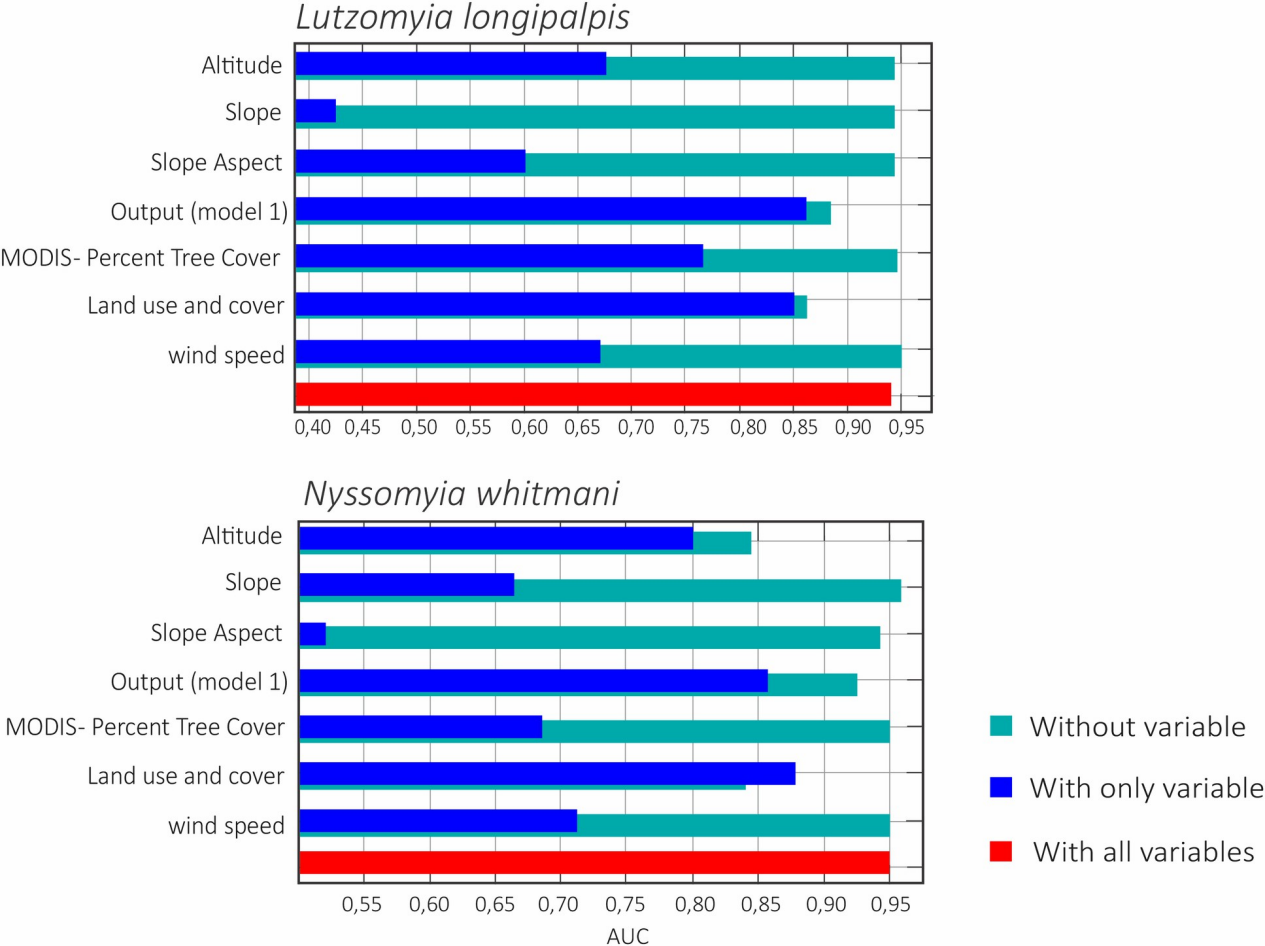
Influence of the environmental variables through the analysis of *Jackknife* test for the predicted environmental suitability of *Lu*. *longipalpis* and *Ny*. *whitmani* in the state of Piauí, Brazil. Without variable, model performance without the analyzed variable; With only variable, model performance when the variable is used alone; With all variables, model performance when all variables are used simultaneously.

Also, the climatic suitability (output model 1) and land use and cover variables when analyzed individually, were the ones that greatest contributed to the AUC values of *Lu*. *longipalpis* and *Ny*. *whitmani* models (Fig 4). The slope and slope aspect variables showed low influence on the models of *Lu*. *longipalpis* and *Ny*. *whitmani*, respectively (Fig 4).

### Incidence of leishmaniasis

**Fig 5:**
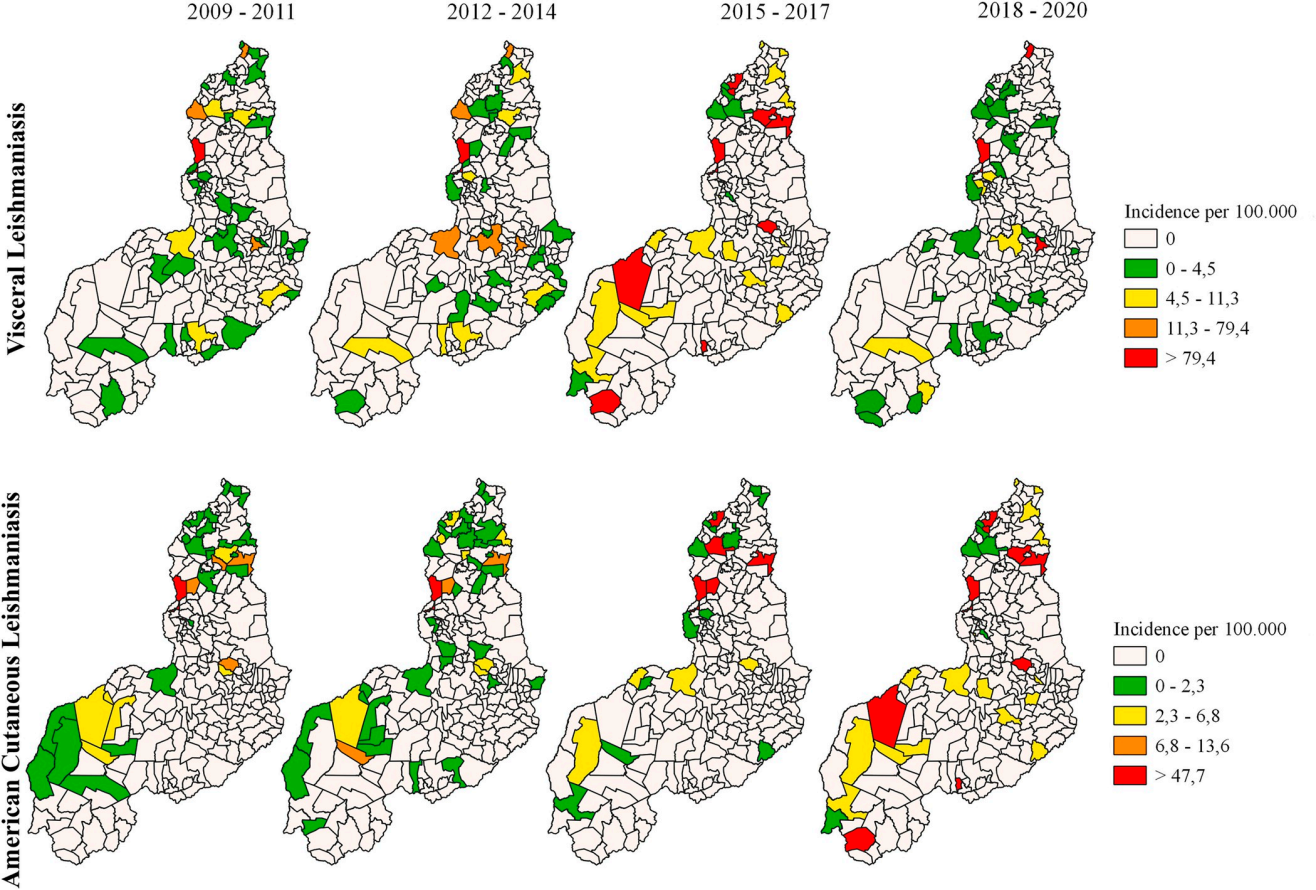
Average incidence rate of VL and ACL by municipality in the state of Piauí, between 2009 and 2020. Available at: https://www.ibge.gov.br/geociencias/downloads- geociencias.html

Throughout the entire study period (2009 to 2020), 140 out of the 224 municipalities (62.50%) did not notify any case of VL. Between 2009 and 2011, the incidence of the disease was considered low with rates ranging from 0 to 4.5 in 29 municipalities (12.95%), with the exception of Picos (located in the center-south of Piauí), Miguel Alves and Parnaiba (in the north) which had medium incidence rates ranging from 11.3 to 79.4; besides the capital Teresina with alarming rates above 79.4/100,000 inhabitants in the same period. Similar pattern of VL incidence was repeated over the other periods, with the exception of the time interval corresponding to 2015–2017, which presented 9 municipalities with alarming incidences above 79.4, namely: Corrente, Anísio de Abreu and Uruçuí (south of the state) and Inhuma, Teresina, Pedro II, Piripiri, São João do Arraial and Luzilândia (in the north region) (Fig 5).

Regarding human ACL, between 2009 and 2020 there were no cases in 138 (61.61%) municipalities. Between 2009 and 2011, 21 municipalities were categorized with low incidence ranging from 0 to 2.3. In the same period the municipalities of Inhuma (in the center of the state), Altos, Capitão de Campos and Pedro II (in the northregion) showed medium rates ranging from 6.8 to 13.6, and only the capital Teresina showed alarming rates of incidence above 47.7/100,000 inhabitants. This pattern of incidence distribution was repeated in the period corresponding to 2012 to 2014. However, there was an increase in the number of municipalities with alarming incidences above 47.7/100,000 inhabitants in the years after 2014. The municipalities with increased ACL incidences between 2015 and 2017 were Teresina, Altos, Pedro II, Batalha and Luzilândia (all in the north of the state), in addition to those who had an increase in notification records in the period between 2018 and 2020, as Corrente and Uruçuí (in the south), Anísio de Abreu (in the extreme south), Inhuma (in the center-north) and Teresina, Pedro II, Piripiri, São João do Arraial and Luzilândia (in the north region) (Fig 5).

### Association between disease incidence and vector suitability

The Spearman correlation between the suitability of vectors and the incidence of VL and ACL at the municipal level was not significant (S2 Table and S7 Fig). Four out of the 11 municipalities with VL incidence above 4.5 cases per 100,000 individuals in the period 2009–2020 demonstrated excellent environmental suitability for *Lu*. *longipalpis* (Floriano, Monsenhor Gil, Parnaíba and Teresina). One municipality (Parnaíba) showed excellent environmental suitability for both vector species (Table 3).

**Table 3:** Environmental suitability for the presence of *Lu*. *longipalpis* and *Ny*. *whitmani* within the municipalities with high incidence rates of VL and ACL between 2009 to 2020.

	VL				ACL		
Environmental Suitability				Environmental Suitability			
Municipalities	*L*. *l*	*N*. *w*	IM (VL)	Municipalities	*L*. *l*	*N*. *w*	IM (ACL)
Teresina	E	H	16.38	Palmeira do Piauí	H	M	33.14
Miguel Alves	H	H	9.10	Altos	E	H	21.51
Floriano	E	H	8.04	Sebastião Leal	H	M	13.53
Monsenhor Gil	E	H	7.10	Ipiranga do Piauí	H	M	12.67
Parnaíba	E	E	5.90	Pedro II	E	E	12.67
Jurema	M	U	5.22	Inhuma	H	M	12.50
Francisco Macedo	H	U	5.18	Capitão de Campos	E	H	10.17
São Raimundo Nonato	M	U	5.00	Porto Alegre do Piauí	H	M	9.16
Santa Rosa Do Piauí	H	M	4.76	Boa Hora	H	H	8.52
São Pedro Do Piauí	H	H	4.64	São João da Fronteira	E	M	8.22
Pavussu	H	M	4.55	Alvorada Do Gurguéia	H	M	7.62
				Milton Brandão	E	H	7.57
				Nossa Senhora dos Remédios	H	M	6.67
				Uruçuí	H	M	6.51
				Baixa Grande do Ribeiro	E	M	5.67
				Ribeiro Gonçalves	H	M	5.62
				Santa Filomena	M	H	5.33
				Matias Olímpio	H	M	5.31
				Luzilândia	H	H	5.22
				São João do Arraial	H	M	5.15
				Brasileira	H	H	4.98
				Joca Marques	H	M	4.56
				Campo Largo do Piauí	H	M	4.54
				Teresina	E	H	4.50
				Santo Antônio dos Milagres	H	H	3.84
				Lagoa de São Francisco	H	E	3.68
				Manoel Emídio	H	M	3.11
				Bom Princípio do Piauí	E	H	2.94
				Joaquim Pires	H	H	2.89
				Antônio Almeida	H	M	2.62
				Barreiras do Piauí	M	M	2.48
				Barro Duro	H	H	2.37

VL, Visceral Leishmaniasis; ACL, American Cutaneous Leishmaniasis; L. l, Lutzomyia longipalpis; N. w, Nyssomyia whitmani; IM, average incidence (cases per 100,000 habitants); E: excellent; H: high; M: medium; U: unsuitable

In the same period, two out of the 32 municipalities with ACL incidence above 2.3 cases per 100,000 habitants showed excellent environmental suitability for the occurrence of *Ny*. *whitmani* (Lagoa de São Francisco and Pedro II), being Pedro II also excellent for *Lu*. *longipalpis* together with other seven municipalities. High suitability for *Lu*. *longipalpis* was observed in 22 municipalities that presented more than 2.3 ACL cases/100,000 inhabitants between 2009–2020, and in six of them the suitability was also high for the presence of *Ny*. *whitmani* (Barro Duro, Boa Hora, Brasileira, Joaquim Pires, Luzilândia and Santo Antonio dos Milagres). Five municipalities revealed high and excellent environmental suitability for *Ny*. *whitmani* and *Lu*. *longipalpis*, respectively (Altos, Capitão de Campos, Milton Brandão, Teresina and Bom Princípio do Piauí) and only one presented high and medium suitability respectively to *Ny*. *whitmani* and *Lu*. *longipalpis*, the municipality of Santa Filomena (Table 3).

## Discussion

This study is the first in the state of Piauí to generate predictive models of environmental suitability for the occurrence of *Lu*. *longipalpis* and *Ny*. *whitmani* and to associate these sandflies with confirmed human cases of VL and ACL. Similar investigation was conducted in the state of Paraná, south Brazil, demonstrating a strong association between the climatic suitability for *Ny*. *whitmani* and the incidence of ACL in the state [34].

The models generated by MaxEnt reveal that environmental and climatic factors are good predictors of the presence of *Lu*. *longipalpis* and *Ny*. *whitmani*, as previously shown elsewhere [13,32,33,35–38]. However, it is important to consider that by adding data from across South America, the models also include local variations in the relationship between vector populations and microclimatic and environmental conditions. Different subspecies or lineages of *Lu*. *longipalpis* and *Ny*. *whitmani* may exhibit distinct local tolerance and respond differently to environmental changes over time. In addition, factors such as urbanization and environmental degradation can affect the distribution and abundance of these insects, leading to unpredictable changes in areas with the highest frequency of each species [36–39].

In the present study, areas with excellent or high suitability for the first vector species extend throughout the central-north region of the state, namely, in the entire intermediate regions of Teresina and Parnaíba (Fig 3). For *Ny*. *whitmani*, the environmental suitability pattern in both intermediate regions was excellent or high in almost 77% of the municipalities (S1 Table and Fig 3). Although the distribution of collection points showed low precision in most of the records (S6 Fig), this did not affect the final ecological niche models, which demonstrated good predictive powers for both vector species, as was observed for *Nyssomyia intermedia*, the main vector species of *Leishmania* parasites causing ACL in southeastern Brazil [39].

The overall shapes of the highly suitable areas in Piauí agree with previous modelling exercises based on similar methods for both *Lu*. *longipalpis* [38] and *Ny*. *whitmani* [13]. The values of the predicted environmental suitability for *Lu*. *longipalpis* were noticeable higher than those for *Ny*. *whitmani* (Fig 3). This could potentially be explained by the climatic and environmental features of the semiarid region of northeast Brazil, where VL initially emerged in the 1930s in a clear association with *Lu*. *longipalpis*, before its expansion related mainly with the urbanization process [40,41].

Land use and cover, together with climate, were the variables that most contributed to the ecological niche models, in accordance to previous studies about the ecology of both vector species. Multiple field studies have shown that *Ny*. *whitmani* has greater preference for rural areas with primary or secondary vegetation, frequently detected in preserved forests [42,43] and less frequently in urban areas [44–46]. However, it has been reported in anthropic environments, such as domestic animal shelters and close to households [44,47]. In this perspective, *Ny*. *whitmani* can be associated with non-anthropic wild areas and urban areas, since the species can be found in both environments, although with low predisposition to invade households [48].

A contrasting scenario is known for *Lu*. *longipalpis*, which in recent decades acquired high adaptability to urban or peri-urban areas, from northwest to southeast Brazil, with high abundance in totally or partially ruralized areas [40,41]. Land cover has also been previously correlated with the prevalence of canine VL in Teresina [49], a municipality with excellent environmental suitability for *Lu*. *longipalpis* according to our results.

In terms of climate, *Ny*. *whitmani* has been previously associated with annual mean temperature values of 19–24°C [13], and in areas of relatively high altitude and subtropical climate with dry winters [34]. These climatic features can be found in the municipalities of Pedro II and Piripiri, which, in the present study had excellent and high environmental suitability for this vector species, respectively. In a seasonality-focused study in southeast Brazil, *Ny*. *whitmani* was more abundant in the coolest months in comparison with warmer periods [44]. In areas where the primitive vegetation cover is similar to that of Piauí, as the municipalities of Mato Grosso and Mato Grosso do Sul, located in the Brazilian Midwest region, *Ny*. *whitmani* can occur in abundance in different types of vegetation like forest, cerrado and pantanal, including transition zones and seasonal deciduous forest. In addition, as in the southeast, greater amounts of this sandfly are found in the months of milder temperatures [42,50,51].

The seasonal behavior of *Lu*. *longipalpis* is also controlled by temperature, humidity and rainfall, as shown by previous study in Mato Grosso do Sul state, Brazil [52]. The potential distributions of both species are expected to expand in Brazil under climate change scenarios [13,35].

Piauí has a long history of VL transmission and on studies tracking its epidemiology. In 1980, the first VL epidemic was found dominating a wide territorial extension of the state, mainly in the urban area of the capital Teresina [53]. This epidemic occurred largely due to rural exodus and disorderly urbanization in the peripheral areas of the municipalities, stimulating the adaptability of *Lu*. *longipalpis* to anthropized areas. The urbanization scenario in the state has grown over the years, reaching an urbanization rate of over 65% distributed in 900.03 km^2^ of built area with greater density in the central and northern regions of the state [54]. Our results demonstrated that the environmental suitability for the two vectors is excellent or high in several municipalities with high rates of urbanization, namely: Teresina, Floriano, Piripiri and Parnaíba.

According to Chaves and cols. 2022, the municipalities in the intermediate region of Teresina have an average lethality rate for human VL of 6.02%, ranging from 6.33% in 2007 to 4.14% in 2019, with some isolated cases in the south region of the immediate geographical area of Piripiri [55]. In our study these regions were predicted as environmentally suitable for both *Lu*. *longipalpis* and *Ny*. *whitmani*. The territories of the immediate region of Teresina, Amarante-Água Branca-Regeneração and Valença do Piauí, in addition to the entire intermediate region of Parnaíba, represent areas with stable lethality of VL and the possibility of increasing the number of notified cases [55]. Our study showed that the incidence rate of VL was considered medium or high in at least one municipality in each region of the state, mainly between 2015 and 2017 (Fig 5).

In contrast, the epidemiology of ACL in Piauí state is less studied due to the lower incidence level of the disease when compared to VL and consequently, the available resources are invested in other areas of health research, such as treatment and prophylaxis instead of applying funds to epidemiological studies [56]. Furthermore, ACL is more prevalent in rural areas of the state, especially in regions of greater social and economic vulnerabilities which in turn have significant underreporting caused by the lack of resources and infrastructure. Overall, these elements generate limitations in epidemiological surveys such as inaccuracy of information, duplicity of notification and/or errors in filling out the forms that make it difficult to find the exact location of the cases [55,57]. In view of this, our results contribute with the knowledge of ACL spatial distribution in the past decade. Over the years, the incidence of ACL has been related to municipalities with low population and occasionally located far from the capital of the state. These are characteristics of the municipalities of Altos and Pedro II, both located in rural areas and presenting high and excellent environmental suitability for *Ny*. *whitmani*, respectively.

Despite not finding significant correlations between human disease reports and environmental suitability for vectors, our qualitative comparisons (Table 3) reveal interesting patterns that can be used to prioritize surveillance and control actions in selected municipalities.

Municipalities with the highest values of incidence for ACL (Palmeira do Piauí, Altos, Sebastião Leal, Ipiranga do Piauí, Pedro II e Inhuma), had at least medium environmental suitability for *Ny*. *whitmani* (Table 3). This pattern was expected, as the presence of the vectors is required for the transmission of *Leishmania* parasites to human. Despite having high and medium environmental suitability for *Lu*. *longipalpis* and *Ny*. *whitmani* respectively, Palmeira do Piauí and Sebastião Leal present no records of the presence of both sandfly vectors, to the best of our knowledge. As these are municipalities with high incidence of ACL, we recommend local field surveys to confirm the presence of these vector species.

In municipalities such as Luís Correia and Cocal, both located in the immediate region of Parnaíba, the environmental suitability for *Lu*. *longipalpis* and *Ny*. *whitmani* were classified as excellent and high, respectively (S1 Table). However, the incidences of VL (< 4.55 cases/100,000 inhabitants) and ACL (< 2.37 cases/100,000 inhabitants) were low throughout the study period. Given the high probability of occurrence of both vectors in these municipalities, collections should be made to confirm their presences, in addition to complementing entomological surveillance with active search for human leishmaniasis cases.

The single municipality considered entirely unsuitable for *Lu*. *longipalpis* (Sebastião Barros, in the immediate region of Corrente–S1 Table) did not have any report of VL human case, thus indicating that the ecological niche models have also performed well in predicting the absence of this vector species. Another scenario that should be confirmed by local entomological survey.

The southeast of the state (intermediate regions of São Raimundo Nonato and Picos) was considered mostly unsuitable for *Ny*. *whitmani* (S1 Table) despite having few municipalities with ACL records, such as Queimada Nova and Jurema [12]. Given the possibility of the absence of *Ny*. *whitmani* in this region of Piauí (which should be confirmed by field monitoring studies), other potential vectors might be present, such as *Ny*. *intermedia* or *Mg*. *migonei*. These species were previously observed in the neighboring states of Ceará and Pernambuco [20,58–60].

The proposed modeling showed that the central-north region of Piauí, an area with high and excellent environmental suitability for *Lu*. *longipalpis* and medium and high for *Ny*. *whitmani*, comprises municipalities with medium and high incidences of VL (between 4,5–11,3 and > 79,4 cases/100,000 inhabitants) and ACL (between 2,3–6,8 and > 47,7 cases/100,000 inhabitants), mainly between 2015 and 2020 (Fig 5). The south and southeast regions of the state have more homogeneous distribution with the presence of municipalities showing low incidences of both the diseases, between 2009 and 2011 (Fig 5). This can be explained by the low environmental suitability for the presence of both vectors in the south and southeast regions of Piauí.

It is worth mentioning that the results presented here are important for public health in the state of Piauí, as well as for studies aiming to relate the ecology of sandflies and disease transmission. The obtained data can be applied as a basis for exploring the distribution of sandflies species and its connection with leishmaniasis occurrence in other geographical areas of Brazil and in other countries, especially where the aspects of geoenvironmental data are similar to those in the northeastern of Brazil. Besides, the described methodology can be replicated in predictive modeling studies of other pathogen transmitting species.

Our results showed that the modeling of the ecological niche of *Lu*. *longipalpis* and *Ny*. *whitmani*, allows a more detailed analysis of how these species are potentially distributed in the state of Piauí and their association with the incidence of leishmaniasis human cases, presenting the entire northern region of the state as a risk area for transmission of *Leishmania* parasites. The identification of municipalities conducive to the emergence of human cases will allow public health authorities to organize surveillance strategies and entomological control of sandflies vectors. Based on our results, we highlight the importance of the entire center and extreme north of Piauí as risk areas for the transmission of the etiological agents of leishmaniasis, with emphasis on municipalities such as Teresina, Altos, Miguel Alves and Pedro II, as well as those presenting similar socio-environmental characteristics. The municipalities of Luiz Correia, Cocal, Palmeira do Piauí and Sebastião Leal should be targeted for entomological surveys to confirm the presence of *Lu*. *longipalpis* and/or *Ny*. *whitmani*, as they were considered highly suitable for the occurrence of these sandflies vectors. In this sense, it should be reinforced that suitability at the scales analyzed in the present study indicates possibility and the ecological niche models of both vector species should be validated with field monitoring results.

## Supporting information

S1 Fig Territories of the state of Piauí with their divisions into immediate and intermediate geographic regions. Available at: https://www.ibge.gov.br/geociencias/downloads-geociencias.html.
(TIF)

S2 Fig Territory of the state of Piauí showing the location of municipalities by immediate and intermediate regions. A. Intermediate region of Parnaíba (30 municipalities). B. Intermediate region of Teresina (65 municipalities). Available at: https://www.ibge.gov.br/geociencias/downloads-geociencias.html.
(TIF)

S3 Fig Territory of the state of Piauí showing the location of municipalities by immediate and intermediate regions. C. Intermediate region of Floriano (28 municipalities). D. Intermediate region of Picos (58 municipalities). Available at: https://www.ibge.gov.br/geociencias/downloads-geociencias.html.
(TIF)

S4 Fig Territory of the state of Piauí showing the location of municipalities by immediate and intermediate regions. E. Intermediate region of Corrente-Bom Jesus (22 municipalities). F. Intermediate region of São Raimundo Nonato (21 municipalities). Available at: https://www.ibge.gov.br/geociencias/downloads-geociencias.html.
(TIFF)

S5 Fig Output (model 1) generated for climate variables. Geographic distribution of sandfly vectors of the causative agents of VL and ACL predicted by modeling based on environmental suitability for South America. AUC: Area Under Curve. Available at: https://www.ibge.gov.br/geociencias/informacoes-ambientais/climatologia/15817-%20182clima.html?=&t=downloads.
(TIF)

S6 Fig Spatial precision of the distribution of *Lutzomyia longipalpis* and *Nyssomyia whitmani* sandfly vectors in the state of Piauí. Points represent known occurrence records classified according to their spatial precision. Available at: https://www.ibge.gov.br/geociencias/downloads-geociencias.html.
(TIF)

S7 Fig Association between disease incidence and vector suitability. Scatterplots of each combination—VL/*Lu*. *longipalpis*, VL/*Ny*. *whitmani*, ACL/*Lu*. *longipalpis*, ACL/*Ny*. *whitmani*. VL, Visceral Leishmaniasis; ACL, American Cutaneous Leishmaniasis.
(TIF)

S1 Table Environmental Suitability for *Lutzomyia longipalpis* and *Nyssomyia whitmani* in the state of Piauí by municipality. Average value of environmental suitability within the limits of each municipality in the state of Piauí and suitability classification.
(DOCX)

S2 Table Spearman’s correlation coefficients. Correlation between vector suitability and VL and ACL incidences at the municipality level.
(DOCX)
