# Supplementary figures and images for: Association between the potential distribution of Lutzomyia longipalpis and Nyssomyia whitmani and leishmaniasis incidence in Piauí State, Brazil
Source: PLoS Negl Trop Dis. 2023 Jun 5;17(6):e0011388. doi: 10.1371/journal.pntd.0011388 (PMC10270596; doi:10.1371/journal.pntd.0011388)

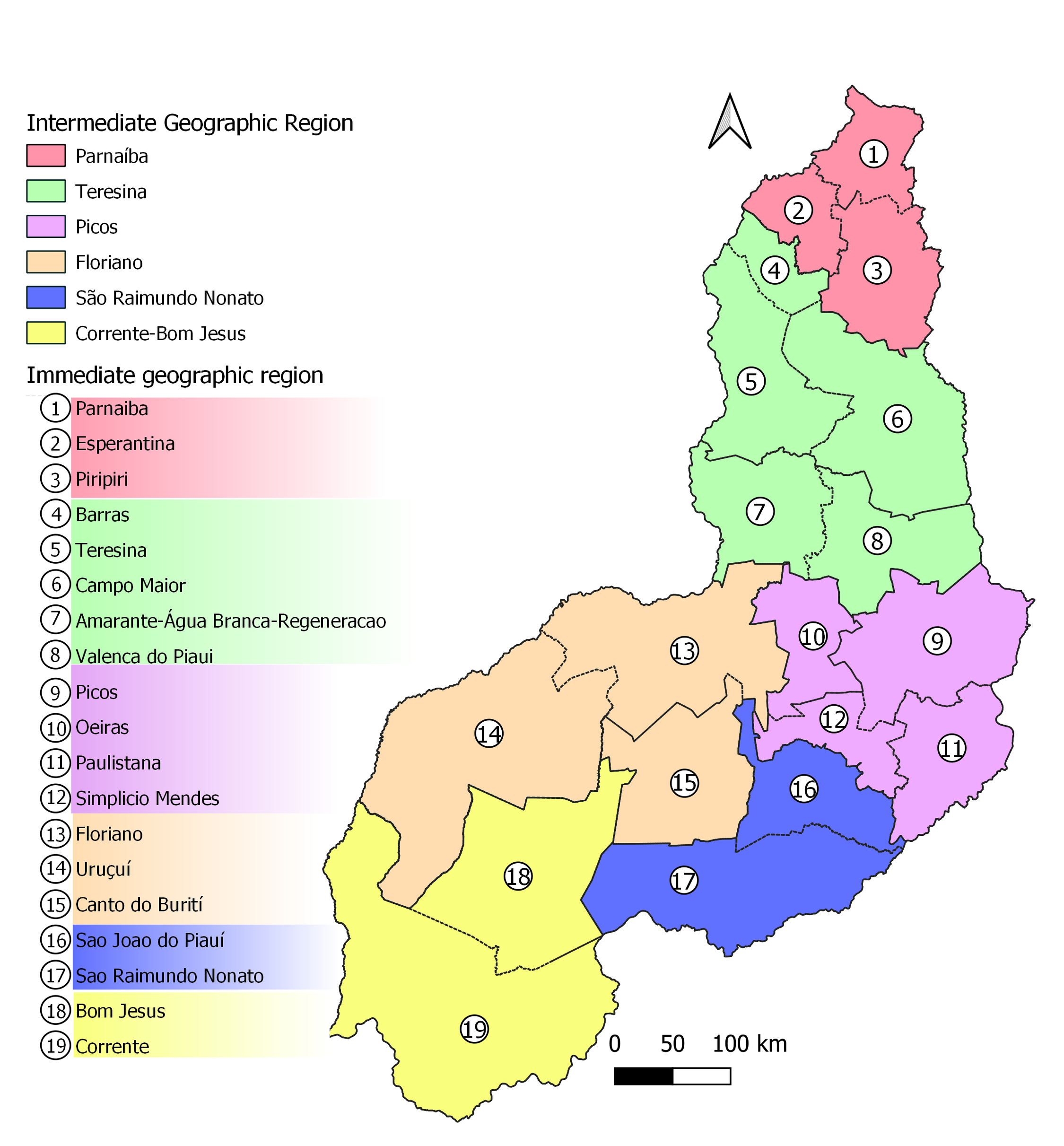

Supplement: S1 Fig — Available at: https://www.ibge.gov.br/geociencias/downloads-geociencias.html. (TIF) [file pntd.0011388.s001.tif]

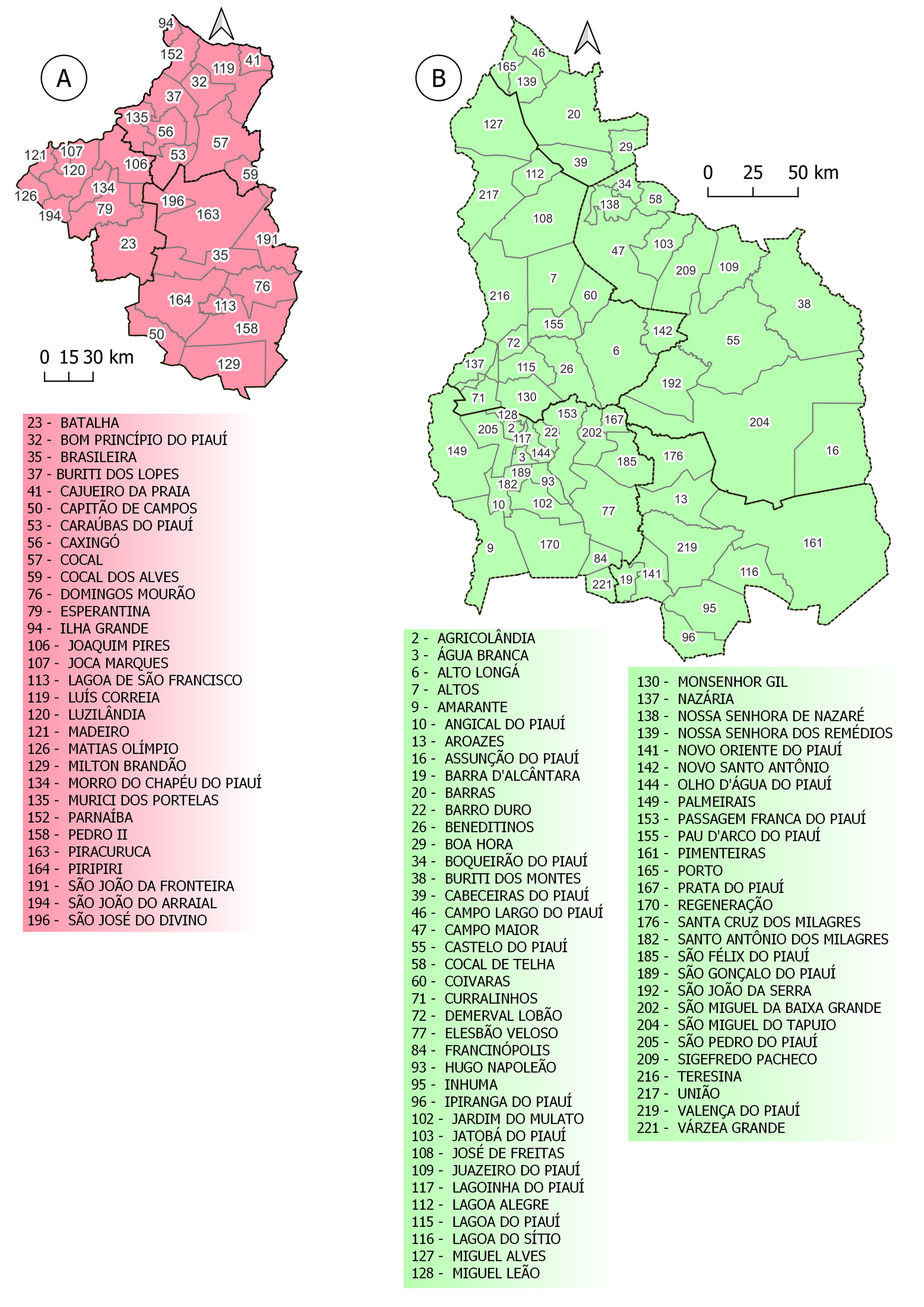

Supplement: S2 Fig — A. Intermediate region of Parnaíba (30 municipalities). B. Intermediate region of Teresina (65 municipalities). Available at: https://www.ibge.gov.br/geociencias/downloads-geociencias.html. (TIF) [file pntd.0011388.s002.tif]

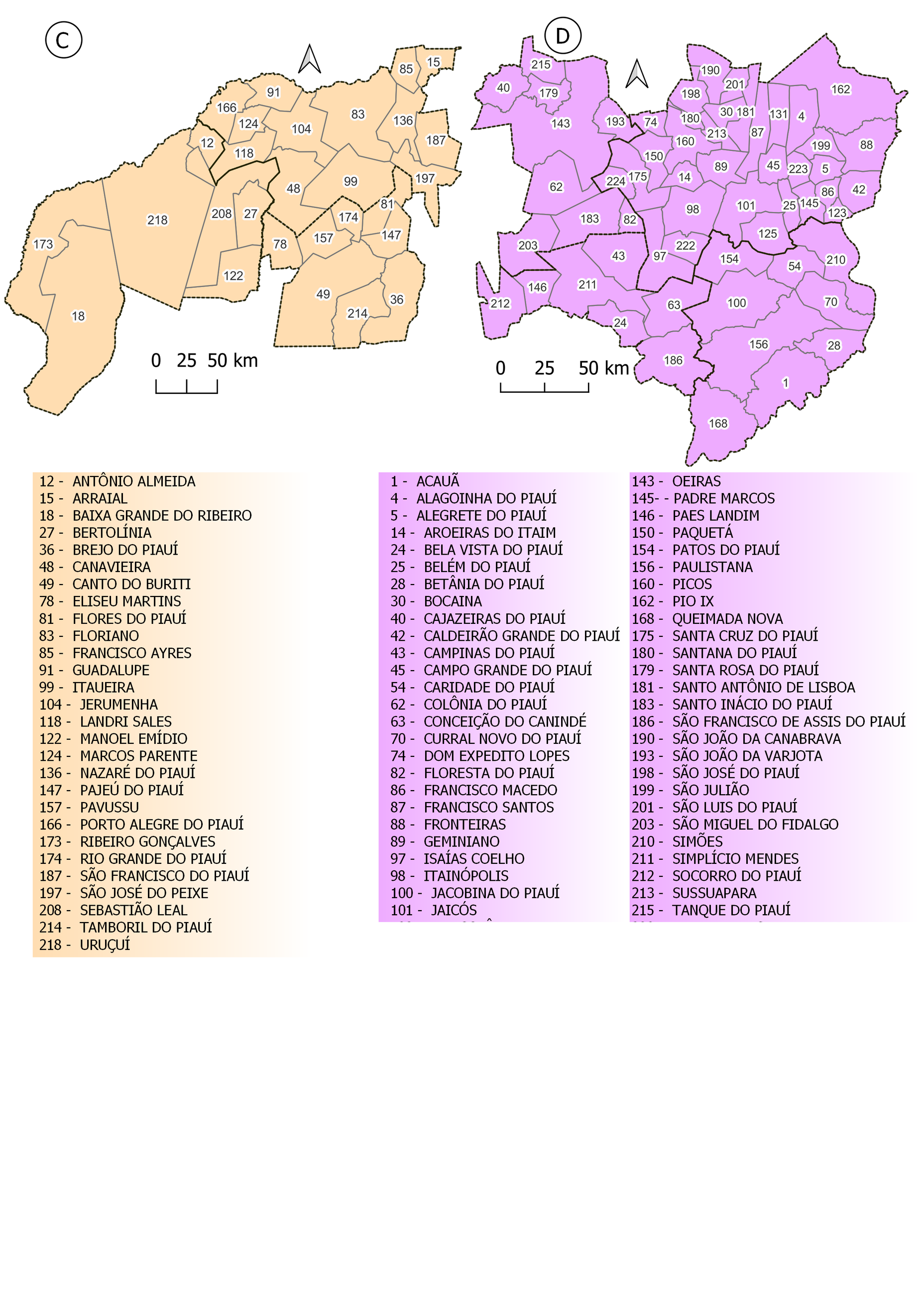

Supplement: S3 Fig — C. Intermediate region of Floriano (28 municipalities). D. Intermediate region of Picos (58 municipalities). Available at: https://www.ibge.gov.br/geociencias/downloads-geociencias.html. (TIF) [file pntd.0011388.s003.tif]

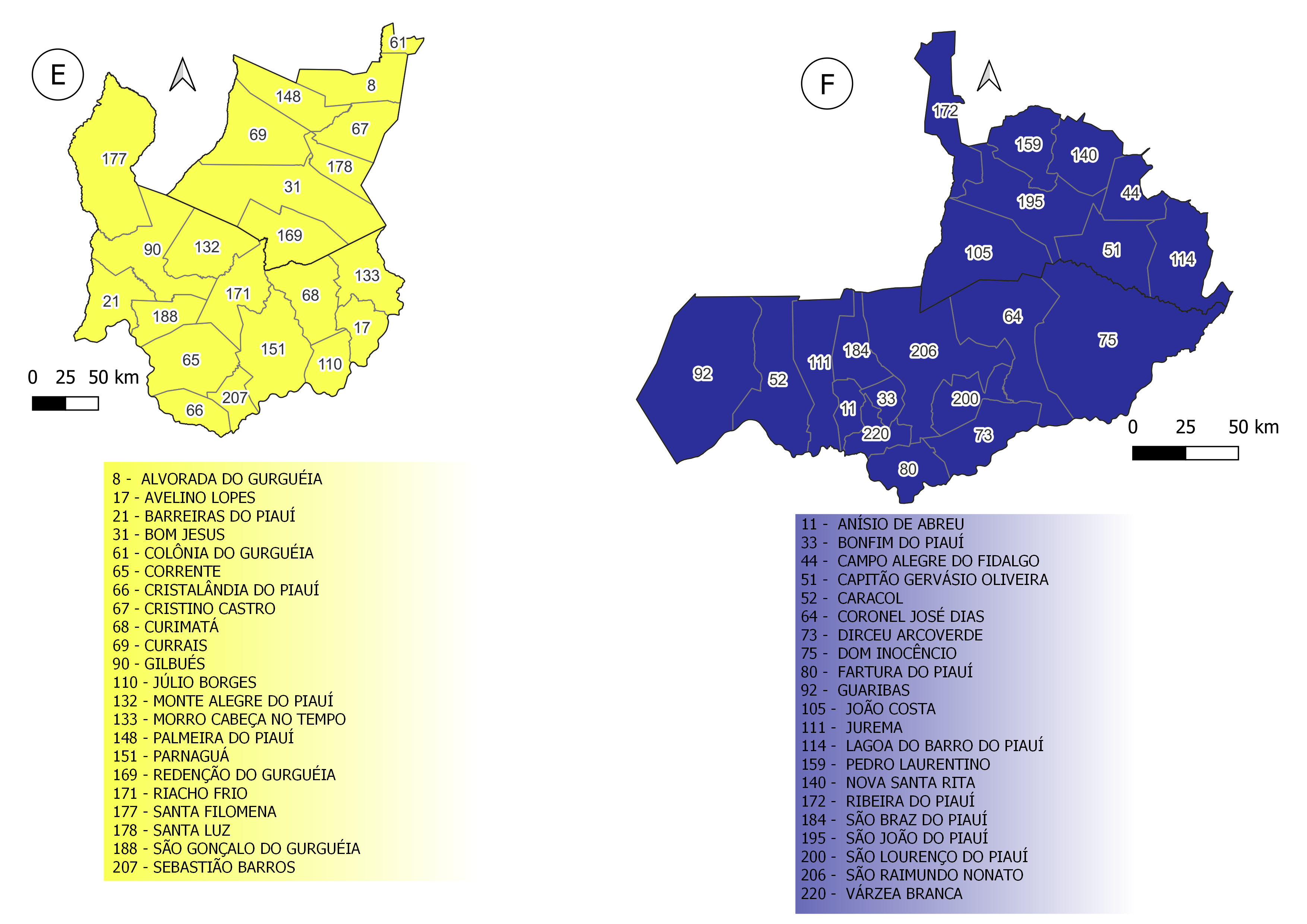

Supplement: S4 Fig — E. Intermediate region of Corrente-Bom Jesus (22 municipalities). F. Intermediate region of São Raimundo Nonato (21 municipalities). Available at: https://www.ibge.gov.br/geociencias/downloads-geociencias.html. (TIFF) [file pntd.0011388.s004.tiff]

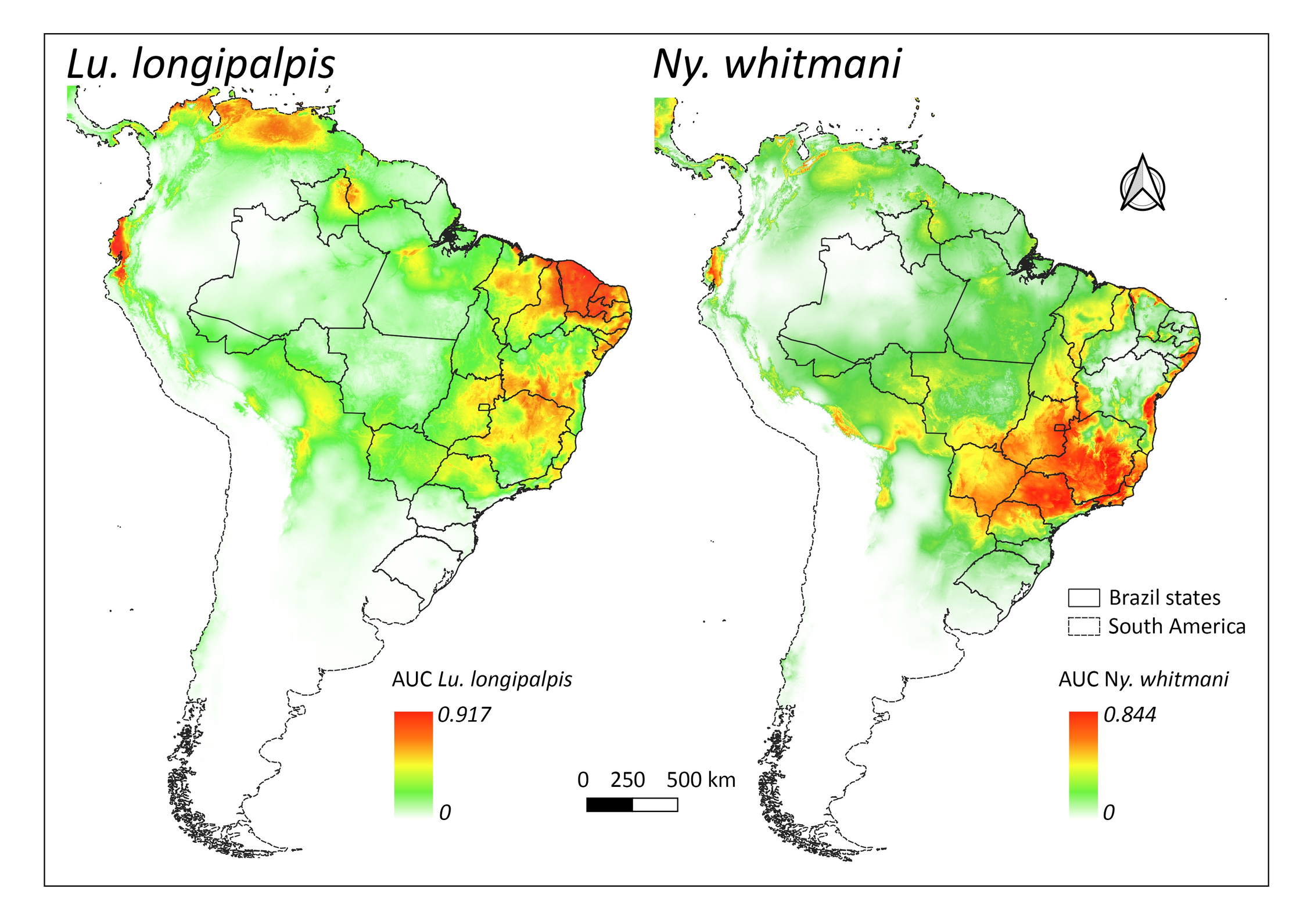

Supplement: S5 Fig — Geographic distribution of sandfly vectors of the causative agents of VL and ACL predicted by modeling based on environmental suitability for South America. AUC: Area Under Curve. Available at: https://www.ibge.gov.br/geociencias/informacoes-ambientais/climatologia/15817-%20182clima.html?=&t=downloads. (TIF) [file pntd.0011388.s005.tif]

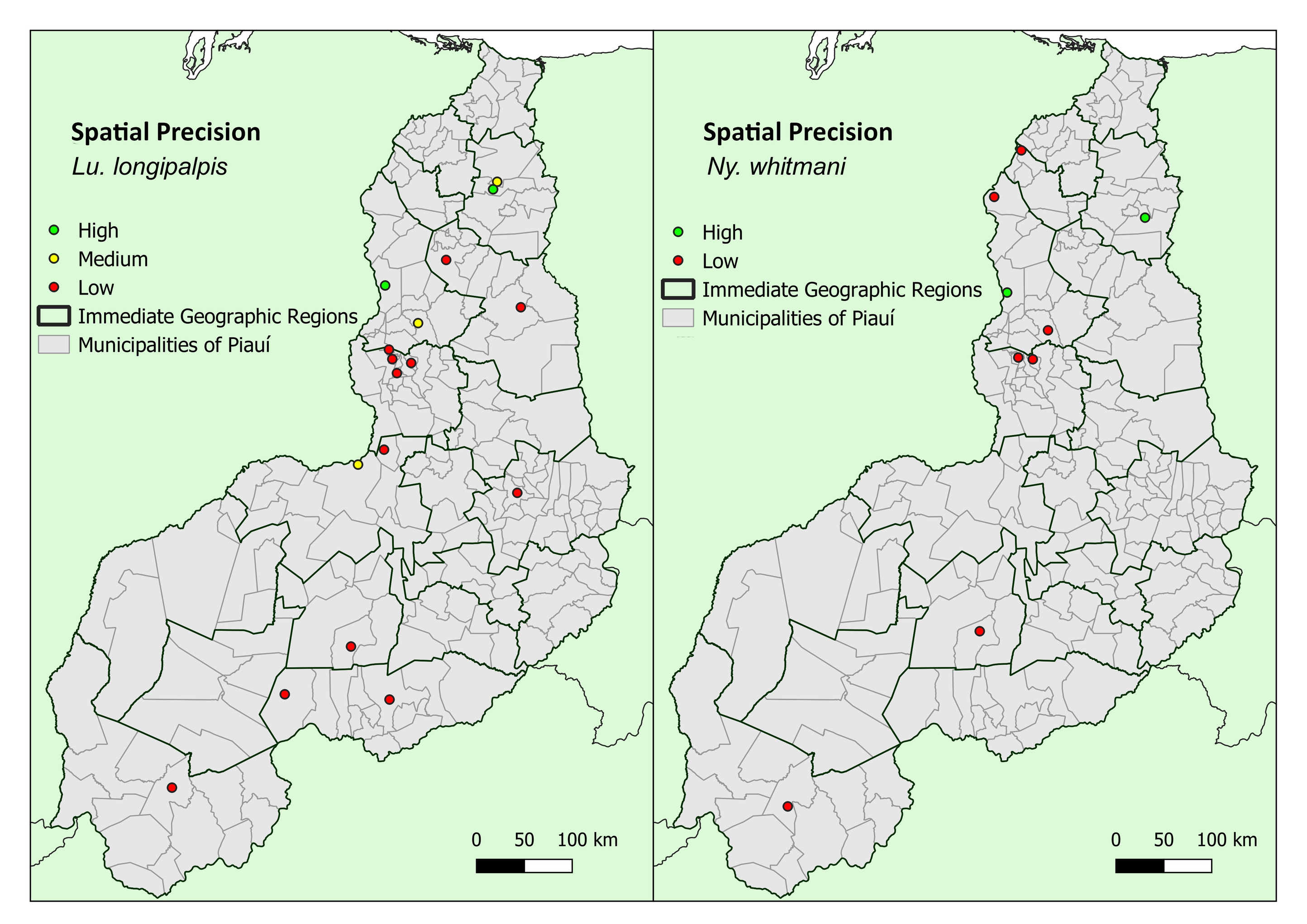

Supplement: S6 Fig — Points represent known occurrence records classified according to their spatial precision. Available at: https://www.ibge.gov.br/geociencias/downloads-geociencias.html. (TIF) [file pntd.0011388.s006.tif]

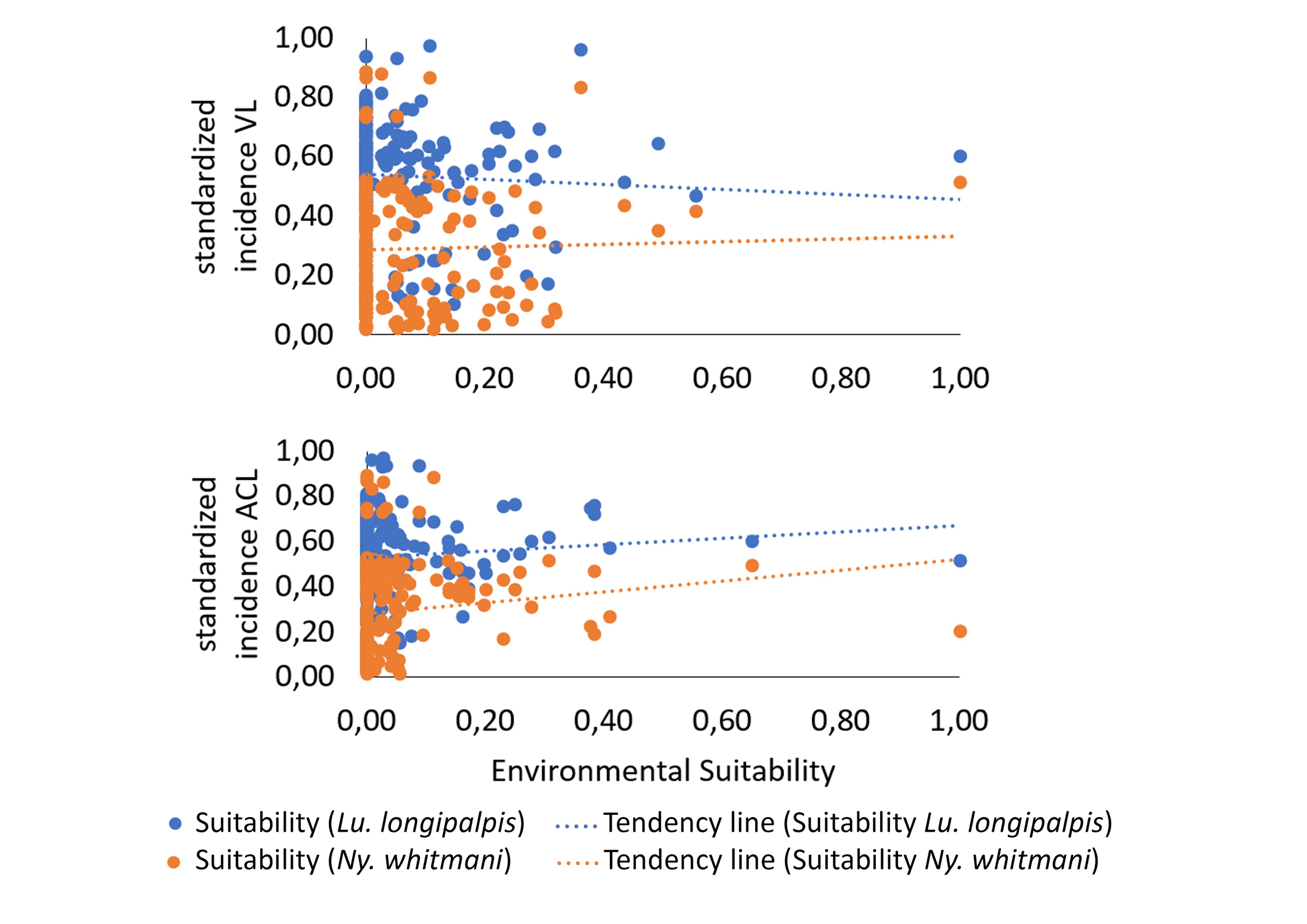

Supplement: S7 Fig — Scatterplots of each combination—VL/Lu. longipalpis, VL/Ny. whitmani, ACL/Lu. longipalpis, ACL/Ny. whitmani. VL, Visceral Leishmaniasis; ACL, American Cutaneous Leishmaniasis. (TIF) [file pntd.0011388.s007.tif]
