# Supplementary material for: Association between the potential distribution of Lutzomyia longipalpis and Nyssomyia whitmani and leishmaniasis incidence in Piauí State, Brazil
Source: PLoS Negl Trop Dis. 2023 Jun 5;17(6):e0011388. doi: 10.1371/journal.pntd.0011388 (PMC10270596; doi:10.1371/journal.pntd.0011388)
Supplement: S1 Table — Average value of environmental suitability within the limits of each municipality in the state of Piauí and suitability classification. (DOCX) [file pntd.0011388.s008.docx]

| **S1Table. Environmental Suitability for *Lutzomyia longipalpis* and *Nyssomyia whitmani* in the state of Piauí, by municipality.** Average value of environmental suitability within the limits of each municipality in the state of Piauí and suitability classification. | | | | | | |
| --- | --- | --- | --- | --- | --- | --- |
| **Geographic Region** | | **Municipalities** | **Environmental Suitability** | | | |
|  |  |  | ***Lu. longipalpis*** | | ***Ny. whitmani*** | |
| **Intermediate** | **Immediate** |  | **Average** | **Classification** | **Average** | **Classification** |
| Parnaíba | Esperantina | Batalha | 0.50 | high | 0.42 | high |
| Parnaíba | Esperantina | Esperantina | 0.51 | high | 0.39 | medium |
| Parnaíba | Esperantina | Joaquim Pires | 0.70 | high | 0.50 | high |
| Parnaíba | Esperantina | Joca Marques | 0.58 | high | 0.39 | medium |
| Parnaíba | Esperantina | Luzilândia | 0.56 | high | 0.41 | high |
| Parnaíba | Esperantina | Madeiro | 0.54 | high | 0.38 | medium |
| Parnaíba | Esperantina | Matias Olímpio | 0.47 | high | 0.36 | medium |
| Parnaíba | Esperantina | Morro Do Chapéu Do Piauí | 0.55 | high | 0.39 | medium |
| Parnaíba | Esperantina | São João Do Arraial | 0.48 | high | 0.36 | medium |
| Parnaíba | Esperantina | Antônio Almeida | 0.58 | high | 0.34 | medium |
| Parnaíba | Esperantina | Arraial | 0.62 | high | 0.49 | high |
| Parnaíba | Parnaíba | Bom Princípio Do Piauí | 0.94 | excellent | 0.73 | high |
| Parnaíba | Parnaíba | Buriti Dos Lopes | 0.82 | excellent | 0.88 | excellent |
| Parnaíba | Parnaíba | Cajueiro Da Praia | 0.94 | excellent | 0.75 | high |
| Parnaíba | Parnaíba | Caraúbas Do Piauí | 0.73 | high | 0.51 | high |
| Parnaíba | Parnaíba | Caxingó | 0.79 | excellent | 0.87 | excellent |
| Parnaíba | Parnaíba | Cocal | 0.79 | excellent | 0.45 | high |
| Parnaíba | Parnaíba | Cocal Dos Alves | 0.79 | excellent | 0.38 | medium |
| Parnaíba | Parnaíba | Ilha Grande | 0.98 | excellent | 0.86 | excellent |
| Parnaíba | Parnaíba | Luís Correia | 0.93 | excellent | 0.52 | high |
| Parnaíba | Parnaíba | Murici Dos Portelas | 0.79 | excellent | 0.89 | excellent |
| Parnaíba | Parnaíba | Parnaíba | 0.96 | excellent | 0.84 | excellent |
| Parnaíba | Piripiri | Brasileira | 0.67 | high | 0.49 | high |
| Parnaíba | Piripiri | Piripiri | 0.61 | high | 0.50 | high |
| Parnaíba | Piripiri | Capitão De Campos | 0.84 | excellent | 0.52 | high |
| Parnaíba | Piripiri | Domingos Mourão | 0.78 | excellent | 0.37 | medium |
| Parnaíba | Piripiri | Lagoa De São Francisco | 0.69 | high | 0.89 | excellent |
| Parnaíba | Piripiri | Milton Brandão | 0.76 | excellent | 0.43 | high |
| Parnaíba | Piripiri | Pedro II | 0.76 | excellent | 0.88 | excellent |
| Parnaíba | Piripiri | Piracuruca | 0.71 | high | 0.48 | high |
| Parnaíba | Piripiri | São João Da Fronteira | 0.77 | excellent | 0.39 | medium |
| Parnaíba | Piripiri | São José Do Divino | 0.61 | high | 0.46 | high |
| Teresina | Amarante-Água Branca-Regeneração | Agricolândia | 0.49 | high | 0.42 | high |
| Teresina | Amarante-Água Branca-Regeneração | Água Branca | 0.47 | high | 0.42 | high |
| Teresina | Amarante-Água Branca-Regeneração | Amarante | 0.58 | high | 0.48 | high |
| Teresina | Amarante-Água Branca-Regeneração | Angical Do Piauí | 0.53 | high | 0.45 | high |
| Teresina | Amarante-Água Branca-Regeneração | Barro Duro | 0.50 | high | 0.42 | high |
| Teresina | Amarante-Água Branca-Regeneração | Hugo Napoleão | 0.52 | high | 0.43 | high |
| Teresina | Amarante-Água Branca-Regeneração | Jardim Do Mulato | 0.55 | high | 0.44 | high |
| Teresina | Amarante-Água Branca-Regeneração | Lagoinha Do Piauí | 0.47 | high | 0.42 | high |
| Teresina | Amarante-Água Branca-Regeneração | Olho D'água Do Piauí | 0.49 | high | 0.42 | high |
| Teresina | Amarante-Água Branca-Regeneração | Palmeirais | 0.55 | high | 0.45 | high |
| Teresina | Amarante-Água Branca-Regeneração | Passagem Franca Do Piauí | 0.54 | high | 0.44 | high |
| Teresina | Amarante-Água Branca-Regeneração | Regeneração | 0.58 | high | 0.47 | high |
| Teresina | Amarante-Água Branca-Regeneração | Santo Antônio Dos Milagres | 0.51 | high | 0.44 | high |
| Teresina | Amarante-Água Branca-Regeneração | São Gonçalo Do Piauí | 0.50 | high | 0.43 | high |
| Teresina | Amarante-Água Branca-Regeneração | São Pedro Do Piauí | 0.53 | high | 0.43 | high |
| Teresina | Amarante-Água Branca-Regeneração | Miguel Leão | 0.51 | high | 0.43 | high |
| Teresina | Amarante-Água Branca-Regeneração | Elesbão Veloso | 0.60 | high | 0.46 | high |
| Teresina | Amarante-Água Branca-Regeneração | Francinópolis | 0.65 | high | 0.48 | high |
| Teresina | Amarante-Água Branca-Regeneração | Prata Do Piauí | 0.64 | high | 0.51 | high |
| Teresina | Amarante-Água Branca-Regeneração | São Félix Do Piauí | 0.61 | high | 0.49 | high |
| Teresina | Amarante-Água Branca-Regeneração | São Miguel Da Baixa Grande | 0.58 | high | 0.46 | high |
| Teresina | Amarante-Água Branca-Regeneração | Várzea Grande | 0.68 | high | 0.47 | high |
| Teresina | Barras | Barras | 0.48 | high | 0.41 | high |
| Teresina | Barras | Boa Hora | 0.55 | high | 0.47 | high |
| Teresina | Barras | Cabeceiras Do Piauí | 0.53 | high | 0.48 | high |
| Teresina | Barras | Campo Largo Do Piauí | 0.46 | high | 0.38 | medium |
| Teresina | Barras | Nossa Senhora Dos Remédios | 0.46 | high | 0.39 | medium |
| Teresina | Campo Maior | Assunção Do Piauí | 0.80 | excellent | 0.29 | medium |
| Teresina | Campo Maior | Boqueirão Do Piauí | 0.60 | high | 0.50 | high |
| Teresina | Campo Maior | Buriti Dos Montes | 0.81 | excellent | 0.30 | medium |
| Teresina | Campo Maior | Campo Maior | 0.60 | high | 0.52 | high |
| Teresina | Campo Maior | Castelo Do Piauí | 0.78 | excellent | 0.39 | medium |
| Teresina | Campo Maior | Cocal De Telha | 0.63 | high | 0.52 | high |
| Teresina | Campo Maior | Jatobá Do Piauí | 0.64 | high | 0.53 | high |
| Teresina | Campo Maior | Juazeiro Do Piauí | 0.76 | excellent | 0.43 | high |
| Teresina | Campo Maior | Nossa Senhora De Nazaré | 0.58 | high | 0.52 | high |
| Teresina | Campo Maior | São João Da Serra | 0.76 | excellent | 0.44 | high |
| Teresina | Campo Maior | São Miguel Do Tapuio | 0.80 | excellent | 0.30 | medium |
| Teresina | Campo Maior | Sigefredo Pacheco | 0.68 | high | 0.74 | high |
| Teresina | Teresina | Miguel Alves | 0.47 | high | 0.42 | high |
| Teresina | Teresina | Alto Longá | 0.65 | high | 0.74 | high |
| Teresina | Teresina | Novo Santo Antônio | 0.71 | high | 0.75 | high |
| Teresina | Teresina | Altos | 0.80 | excellent | 0.41 | high |
| Teresina | Teresina | Beneditinos | 0.59 | high | 0.50 | high |
| Teresina | Teresina | Coivaras | 0.59 | high | 0.51 | high |
| Teresina | Teresina | Curralinhos | 0.53 | high | 0.44 | high |
| Teresina | Teresina | Demerval Lobão | 0.62 | high | 0.52 | high |
| Teresina | Teresina | José De Freitas | 0.57 | high | 0.51 | high |
| Teresina | Teresina | Lagoa Alegre | 0.52 | high | 0.47 | high |
| Teresina | Teresina | Lagoa Do Piauí | 0.57 | high | 0.49 | high |
| Teresina | Teresina | Monsenhor Gil | 0.81 | excellent | 0.44 | high |
| Teresina | Teresina | Nazária | 0.56 | high | 0.48 | high |
| Teresina | Teresina | Pau D'arco Do Piauí | 0.61 | high | 0.51 | high |
| Teresina | Teresina | Teresina | 0.90 | excellent | 0.52 | high |
| Teresina | Teresina | União | 0.52 | high | 0.48 | high |
| Teresina | Valença do Piauí | Ipiranga Do Piauí | 0.72 | high | 0.19 | medium |
| Teresina | Valença do Piauí | Aroazes | 0.72 | high | 0.40 | medium |
| Teresina | Valença do Piauí | Barra D'alcântara | 0.70 | high | 0.43 | high |
| Teresina | Valença do Piauí | Inhuma | 0.75 | high | 0.23 | medium |
| Teresina | Valença do Piauí | Lagoa Do Sítio | 0.79 | excellent | 0.25 | medium |
| Teresina | Valença do Piauí | Novo Oriente Do Piauí | 0.71 | high | 0.37 | medium |
| Teresina | Valença do Piauí | Pimenteiras | 0.77 | excellent | 0.22 | medium |
| Teresina | Valença do Piauí | Santa Cruz Dos Milagres | 0.73 | high | 0.43 | high |
| Teresina | Valença do Piauí | Valença Do Piauí | 0.74 | high | 0.34 | medium |
| Picos | Oeiras | Floresta Do Piauí | 0.51 | high | 0.10 | unsuitable |
| Picos | Oeiras | Santo Inácio Do Piauí | 0.44 | high | 0.08 | unsuitable |
| Picos | Oeiras | São Miguel Do Fidalgo | 0.45 | high | 0.09 | unsuitable |
| Picos | Oeiras | Cajazeiras Do Piauí | 0.67 | high | 0.42 | high |
| Picos | Oeiras | Colônia Do Piauí | 0.59 | high | 0.14 | medium |
| Picos | Oeiras | Oeiras | 0.70 | high | 0.25 | medium |
| Picos | Oeiras | Santa Rosa Do Piauí | 0.69 | high | 0.35 | medium |
| Picos | Oeiras | São João Da Varjota | 0.71 | high | 0.17 | medium |
| Picos | Oeiras | Tanque Do Piauí | 0.68 | high | 0.43 | high |
| Picos | Paulistana | Acauã | 0.24 | medium | 0.03 | unsuitable |
| Picos | Paulistana | Betânia Do Piauí | 0.35 | medium | 0.05 | unsuitable |
| Picos | Paulistana | Caridade Do Piauí | 0.45 | high | 0.06 | unsuitable |
| Picos | Paulistana | Curral Novo Do Piauí | 0.44 | high | 0.06 | unsuitable |
| Picos | Paulistana | Jacobina Do Piauí | 0.38 | medium | 0.06 | unsuitable |
| Picos | Paulistana | Patos Do Piauí | 0.52 | high | 0.09 | unsuitable |
| Picos | Paulistana | Paulistana | 0.28 | medium | 0.04 | unsuitable |
| Picos | Paulistana | Queimada Nova | 0.16 | medium | 0.02 | unsuitable |
| Picos | Paulistana | Simões | 0.57 | high | 0.10 | unsuitable |
| Picos | Picos | Belém Do Piauí | 0.58 | high | 0.08 | unsuitable |
| Picos | Picos | Caldeirão Grande Do Piauí | 0.67 | high | 0.15 | medium |
| Picos | Picos | Campo Grande Do Piauí | 0.61 | high | 0.08 | unsuitable |
| Picos | Picos | Francisco Macedo | 0.62 | high | 0.09 | unsuitable |
| Picos | Picos | Fronteiras | 0.63 | high | 0.09 | unsuitable |
| Picos | Picos | Isaías Coelho | 0.56 | high | 0.14 | medium |
| Picos | Picos | Itainópolis | 0.62 | high | 0.12 | medium |
| Picos | Picos | Jaicós | 0.61 | high | 0.09 | unsuitable |
| Picos | Picos | Marcolândia | 0.66 | high | 0.14 | medium |
| Picos | Picos | Massapê Do Piauí | 0.56 | high | 0.08 | unsuitable |
| Picos | Picos | Padre Marcos | 0.60 | high | 0.08 | unsuitable |
| Picos | Picos | Vera Mendes | 0.62 | high | 0.15 | medium |
| Picos | Picos | Vila Nova Do Piauí | 0.59 | high | 0.07 | unsuitable |
| Picos | Picos | Aroeiras Do Itaim | 0.65 | high | 0.12 | medium |
| Picos | Picos | Bocaina | 0.67 | high | 0.11 | medium |
| Picos | Picos | Dom Expedito Lopes | 0.68 | high | 0.16 | medium |
| Picos | Picos | Geminiano | 0.65 | high | 0.11 | medium |
| Picos | Picos | Paquetá | 0.64 | high | 0.12 | medium |
| Picos | Picos | Picos | 0.68 | high | 0.15 | medium |
| Picos | Picos | Santa Cruz Do Piauí | 0.59 | high | 0.11 | medium |
| Picos | Picos | Santana Do Piauí | 0.70 | high | 0.15 | medium |
| Picos | Picos | São João Da Canabrava | 0.77 | excellent | 0.21 | medium |
| Picos | Picos | São José Do Piauí | 0.74 | high | 0.19 | medium |
| Picos | Picos | São Luis Do Piauí | 0.73 | high | 0.16 | medium |
| Picos | Picos | Sussuapara | 0.67 | high | 0.11 | medium |
| Picos | Picos | Wall Ferraz | 0.55 | high | 0.11 | medium |
| Picos | Picos | Alagoinha Do Piauí | 0.65 | high | 0.10 | unsuitable |
| Picos | Picos | Alegrete Do Piauí | 0.61 | high | 0.08 | unsuitable |
| Picos | Picos | Francisco Santos | 0.64 | high | 0.10 | unsuitable |
| Picos | Picos | Monsenhor Hipólito | 0.65 | high | 0.12 | medium |
| Picos | Picos | Pio Ix | 0.68 | high | 0.13 | medium |
| Picos | Picos | Santo Antônio De Lisboa | 0.67 | high | 0.12 | medium |
| Picos | Picos | São Julião | 0.61 | high | 0.08 | unsuitable |
| Picos | Simplício Mendes | Bela Vista Do Piauí | 0.31 | medium | 0.06 | unsuitable |
| Picos | Simplício Mendes | Campinas Do Piauí | 0.45 | high | 0.09 | unsuitable |
| Picos | Simplício Mendes | Conceição Do Canindé | 0.45 | high | 0.10 | unsuitable |
| Picos | Simplício Mendes | Paes Landim | 0.36 | medium | 0.07 | unsuitable |
| Picos | Simplício Mendes | São Francisco De Assis Do Piauí | 0.25 | medium | 0.04 | unsuitable |
| Picos | Simplício Mendes | Simplício Mendes | 0.36 | medium | 0.07 | unsuitable |
| Picos | Simplício Mendes | Socorro Do Piauí | 0.36 | medium | 0.07 | unsuitable |
| São Raimundo Nonato | São João do Piauí | Campo Alegre Do Fidalgo | 0.21 | medium | 0.03 | unsuitable |
| São Raimundo Nonato | São João do Piauí | Capitão Gervásio Oliveira | 0.15 | medium | 0.02 | unsuitable |
| São Raimundo Nonato | São João do Piauí | João Costa | 0.18 | medium | 0.04 | unsuitable |
| São Raimundo Nonato | São João do Piauí | Lagoa Do Barro Do Piauí | 0.13 | medium | 0.02 | unsuitable |
| São Raimundo Nonato | São João do Piauí | Nova Santa Rita | 0.25 | medium | 0.05 | unsuitable |
| São Raimundo Nonato | São João do Piauí | Pedro Laurentino | 0.28 | medium | 0.06 | unsuitable |
| São Raimundo Nonato | São João do Piauí | Ribeira Do Piauí | 0.38 | medium | 0.09 | unsuitable |
| São Raimundo Nonato | São João do Piauí | São João Do Piauí | 0.20 | medium | 0.04 | unsuitable |
| São Raimundo Nonato | São Raimundo Nonato | Anísio De Abreu | 0.18 | medium | 0.04 | unsuitable |
| São Raimundo Nonato | São Raimundo Nonato | Bonfim Do Piauí | 0.13 | medium | 0.03 | unsuitable |
| São Raimundo Nonato | São Raimundo Nonato | Caracol | 0.34 | medium | 0.10 | unsuitable |
| São Raimundo Nonato | São Raimundo Nonato | Coronel José Dias | 0.16 | medium | 0.03 | unsuitable |
| São Raimundo Nonato | São Raimundo Nonato | Dirceu Arcoverde | 0.15 | medium | 0.03 | unsuitable |
| São Raimundo Nonato | São Raimundo Nonato | Dom Inocêncio | 0.14 | medium | 0.02 | unsuitable |
| São Raimundo Nonato | São Raimundo Nonato | Fartura Do Piauí | 0.14 | medium | 0.03 | unsuitable |
| São Raimundo Nonato | São Raimundo Nonato | Guaribas | 0.44 | high | 0.13 | medium |
| São Raimundo Nonato | São Raimundo Nonato | Jurema | 0.30 | medium | 0.08 | unsuitable |
| São Raimundo Nonato | São Raimundo Nonato | São Braz Do Piauí | 0.25 | medium | 0.07 | unsuitable |
| São Raimundo Nonato | São Raimundo Nonato | São Lourenço Do Piauí | 0.14 | medium | 0.03 | unsuitable |
| São Raimundo Nonato | São Raimundo Nonato | São Raimundo Nonato | 0.17 | medium | 0.04 | unsuitable |
| São Raimundo Nonato | São Raimundo Nonato | Várzea Branca | 0.15 | medium | 0.03 | unsuitable |
| Corrente-Bom Jesus | Bom Jesus | Alvorada Do Gurguéia | 0.54 | high | 0.17 | medium |
| Corrente-Bom Jesus | Bom Jesus | Barreiras Do Piauí | 0.18 | medium | 0.32 | medium |
| Corrente-Bom Jesus | Bom Jesus | Bom Jesus | 0.42 | high | 0.21 | medium |
| Corrente-Bom Jesus | Bom Jesus | Cristino Castro | 0.52 | high | 0.14 | medium |
| Corrente-Bom Jesus | Bom Jesus | Currais | 0.46 | high | 0.21 | medium |
| Corrente-Bom Jesus | Bom Jesus | Gilbués | 0.25 | medium | 0.30 | medium |
| Corrente-Bom Jesus | Bom Jesus | Monte Alegre Do Piauí | 0.30 | medium | 0.23 | medium |
| Corrente-Bom Jesus | Bom Jesus | Palmeira Do Piauí | 0.52 | high | 0.21 | medium |
| Corrente-Bom Jesus | Bom Jesus | Redenção Do Gurguéia | 0.35 | medium | 0.23 | medium |
| Corrente-Bom Jesus | Bom Jesus | Santa Luz | 0.54 | high | 0.19 | medium |
| Corrente-Bom Jesus | Bom Jesus | Santa Filomena | 0.27 | medium | 0.42 | high |
| Corrente-Bom Jesus | Bom Jesus | Colônia Do Gurguéia | 0.51 | high | 0.15 | medium |
| Corrente-Bom Jesus | Corrente | Avelino Lopes | 0.20 | medium | 0.10 | unsuitable |
| Corrente-Bom Jesus | Corrente | Corrente | 0.16 | medium | 0.24 | medium |
| Corrente-Bom Jesus | Corrente | Cristalândia Do Piauí | 0.12 | medium | 0.23 | medium |
| Corrente-Bom Jesus | Corrente | Curimatá | 0.27 | medium | 0.16 | medium |
| Corrente-Bom Jesus | Corrente | Júlio Borges | 0.17 | medium | 0.17 | medium |
| Corrente-Bom Jesus | Corrente | Morro Cabeça No Tempo | 0.29 | medium | 0.11 | medium |
| Corrente-Bom Jesus | Corrente | Parnaguá | 0.16 | medium | 0.17 | medium |
| Corrente-Bom Jesus | Corrente | Riacho Frio | 0.24 | medium | 0.18 | medium |
| Corrente-Bom Jesus | Corrente | Sebastião Barros | 0.10 | unsuitable | 0.20 | medium |
| Corrente-Bom Jesus | Corrente | São Gonçalo Do Gurguéia | 0.20 | medium | 0.27 | medium |
| Floriano | Uruçuí | Baixa Grande Do Ribeiro | 0.80 | excellent | 0.36 | medium |
| Floriano | Uruçuí | Ribeiro Gonçalves | 0.46 | high | 0.38 | medium |
| Floriano | Uruçuí | Uruçuí | 0.50 | high | 0.32 | medium |
| Floriano | Floriano | Porto | 0.45 | high | 0.39 | medium |
| Floriano | Uruçuí | Bertolínia | 0.63 | high | 0.20 | medium |
| Floriano | Canto do Burití | Eliseu Martins | 0.58 | high | 0.17 | medium |
| Floriano | Floriano | Landri Sales | 0.60 | high | 0.33 | medium |
| Floriano | Uruçuí | Manoel Emídio | 0.58 | high | 0.19 | medium |
| Floriano | Floriano | Marcos Parente | 0.62 | high | 0.29 | medium |
| Floriano | Floriano | Porto Alegre Do Piauí | 0.60 | high | 0.31 | medium |
| Floriano | Uruçuí | Sebastião Leal | 0.57 | high | 0.27 | medium |
| Floriano | Floriano | Canavieira | 0.65 | high | 0.26 | medium |
| Floriano | Canto do Buriti | Flores Do Piauí | 0.57 | high | 0.13 | medium |
| Floriano | Floriano | Floriano | 0.86 | excellent | 0.65 | high |
| Floriano | Floriano | Guadalupe | 0.63 | high | 0.35 | medium |
| Floriano | Floriano | Itaueira | 0.64 | high | 0.25 | medium |
| Floriano | Floriano | Jerumenha | 0.64 | high | 0.36 | medium |
| Floriano | Floriano | Nazaré Do Piauí | 0.69 | high | 0.28 | medium |
| Floriano | Canto do Buriti | Pavussu | 0.60 | high | 0.17 | medium |
| Floriano | Canto do Buriti | Rio Grande Do Piauí | 0.59 | high | 0.16 | medium |
| Floriano | Floriano | São Francisco Do Piauí | 0.70 | high | 0.23 | medium |
| Floriano | Floriano | São José Do Peixe | 0.60 | high | 0.16 | medium |
| Floriano | Floriano | Francisco Ayres | 0.63 | high | 0.48 | high |
| Floriano | Canto do Buriti | Brejo Do Piauí | 0.28 | medium | 0.06 | unsuitable |
| Floriano | Canto do Buriti | Canto Do Buriti | 0.48 | high | 0.13 | medium |
| Floriano | Canto do Buriti | Pajeú Do Piauí | 0.44 | high | 0.09 | unsuitable |
| Floriano | Canto do Buriti | Tamboril Do Piauí | 0.36 | medium | 0.09 | unsuitable |
