# Supplementary material for: Association between the potential distribution of Lutzomyia longipalpis and Nyssomyia whitmani and leishmaniasis incidence in Piauí State, Brazil
Source: PLoS Negl Trop Dis. 2023 Jun 5;17(6):e0011388. doi: 10.1371/journal.pntd.0011388 (PMC10270596; doi:10.1371/journal.pntd.0011388)
Supplement: S2 Table — Correlation between vector suitability and VL and ACL incidences at the municipality level. (DOCX) [file pntd.0011388.s009.docx]

| **S2 Table. Spearman's correlation coefficients.** Correlation between vector suitability and VL and ACL incidences at the municipality level | | |
| --- | --- | --- |
|  | **Environmental suitability**  ***Lu. longipalpis*** | **Environmental suitability**  ***Ny. whitmani*** |
| VL incidence | - 0.095 | - 0.019 |
| P-Value | 0.158 | 0.778 |
| ACL incidence | 0.028 | 0.281 |
| P-Value | 0.673 | 0.380 |
